# Cascade NH_3_ Oxidation and N_2_O Decomposition via Bifunctional Co and Cu Catalysts

**DOI:** 10.1021/acscatal.3c02392

**Published:** 2023-10-12

**Authors:** Xuze Guan, Hiroyuki Asakura, Rong Han, Siyuan Xu, Hao-Xin Liu, Lu Chen, Zhangyi Yao, Jay Hon Cheung Yan, Tsunehiro Tanaka, Yuzheng Guo, Chun-Jiang Jia, Feng Ryan Wang

**Affiliations:** † Department of Chemical Engineering, University College London, Roberts Building, Torrington Place, London WC1E 7JE, U.K.; ‡ Department of Applied Chemistry, Faculty of Science and Engineering, Kindai University 3-4-1, Kowakae, Higashiosaka, Osaka 577-8502, Japan; § Department of Molecular Engineering, Graduate School of Engineering, Kyoto University, Kyotodaigaku Katsura, Nishikyo-ku, Kyoto, Kyoto 615-8510, Japan; ∥ School of Electrical Engineering and Automation, Wuhan University, Wuhan, Hubei 430072, China; ⊥ Key Laboratory for Colloid and Interface Chemistry, Key Laboratory of Special Aggregated Materials, School of Chemistry and Chemical Engineering, Shandong University, Jinan, Shandong 250100, China

**Keywords:** ammonia-selective oxidation, CuO/Co_3_O_4_ catalyst, N_2_O decomposition, metal−support interaction, O_2_ activation

## Abstract

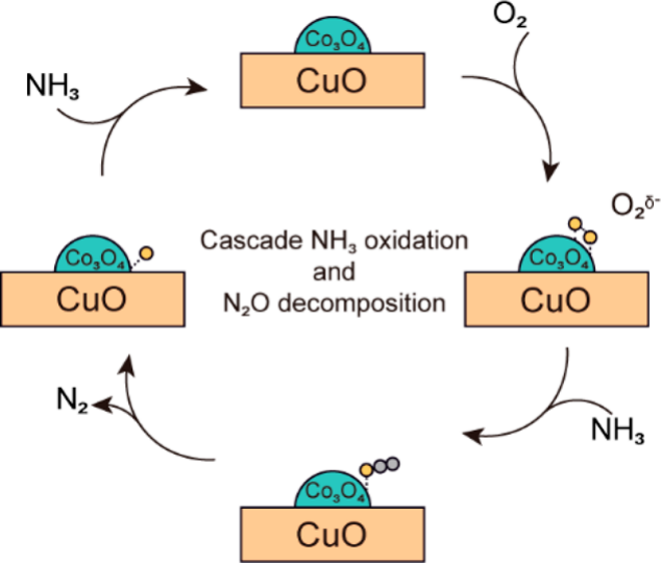

The selective catalytic oxidation of NH_3_ (NH_3_–SCO) to N_2_ is an important reaction for the treatment of diesel engine exhaust. Co_3_O_4_ has the highest activity among non-noble metals but suffers from N_2_O release. Such N_2_O emissions have recently been regulated due to having a 300× higher greenhouse gas effect than CO_2_. Here, we design CuO-supported Co_3_O_4_ as a cascade catalyst for the selective oxidation of NH_3_ to N_2_. The NH_3_–SCO reaction on CuO–Co_3_O_4_ follows a de-N_2_O pathway. Co_3_O_4_ activates gaseous oxygen to form N_2_O. The high redox property of the CuO–Co_3_O_4_ interface promotes the breaking of the N–O bond in N_2_O to form N_2_. The addition of CuO–Co_3_O_4_ to the Pt–Al_2_O_3_ catalyst reduces the full NH_3_ conversion temperature by 50 K and improves the N_2_ selectivity by 20%. These findings provide a promising strategy for reducing N_2_O emissions and will contribute to the rational design and development of non-noble metal catalysts.

## 1 Introduction

Excess ammonia (NH_3_) is needed for selective catalytic reduction (NH_3_–SCR) of harmful nitrogen oxides (NO_*x*_) from diesel vehicle exhaust and power plants.^1−3^ The slip of unreacted NH_3_ can be eliminated by selective catalytic oxidation of NH_3_ (NH_3_–SCO) to nitrogen.^4^ The ideal catalysts for the NH_3_–SCO reaction downstream of NH_3_–SCR should completely convert NH_3_ to N_2_ and H_2_O without the reformation of N-containing pollutants. Co_3_O_4_ is a promising catalyst with comparable activity to replace noble metals.^5−8^ However, Co_3_O_4_ will lead to the release of nitrous oxide (N_2_O), which has a greenhouse gas effect 300× higher than that of CO_2_.^9,10^ In particular, N_2_O emission in the transportation sector is significant, with 56 mg N_2_O/km generated by on-road vehicles.^11^ Without a global mitigation strategy, N_2_O emissions are projected to be nearly double by 2050.^12^ Therefore, N_2_O emissions from diesel engines have been recently regulated and are included in the upcoming EU7 standard. Considering this, the high N_2_O release rate from Co_3_O_4_ makes it unsuitable for NH_3_–SCO.

NH_3_–SCR and NH_3_–SCO catalytic reactions have been extensively studied for decades, but limited attention has been paid to the side reactions of N_2_O formation.^13−17^ Noble metals typically have high NH_3_–SCO activity, but significant N_2_O release at high temperatures cannot be avoided.^18−22^ Gang et al.^23^ found that oxygen dissociation is the rate-controlling step of NH_3_–SCO on a Ag-powder catalyst. The selectivity for N_2_, N_2_O, and NO mainly depends on the surface oxygen coverage and reaction temperature. The nitrogen selectivity increases with the adsorption of NO_*x*_ and N_2_O_*x*_ species, as the oxidation of NH_3_ is inhibited due to the reduced concentration of surface-active O. These results are consistent with studies on Ir-based^24^ and Au-based^25^ catalysts. Karatok et al.^26^ reported that disordered surface atomic O can selectively catalyze N–H bond cleavage, yielding mainly N_2_ products, while the O/Ag(111) surface with higher oxygen coverage shows higher selectivity for N_2_O than N_2_. Therefore, it has been pointed out that blocking oxygen dissociation sites on noble metals can effectively improve N_2_ selectivity, but this also leads to a loss of activity.^6^ Transition metal oxides, such as CuO,^27−32^ Fe_2_O_3_,^33,34^ and V_2_O_5_,^35^ have been reported to have high N_2_ selectivity but lack sufficient activity for practical use. Despite all these efforts, there remains an insufficient understanding of the fundamental methods for achieving high low-temperature activity with less N_2_O emission for NH_3_–SCO.

In this study, we designed a cascade catalyst that uses CuO to help remove the as-formed N_2_O from Co_3_O_4_. Such a 3d metal oxide system showed similar performance to Pt–Al_2_O_3_, which is usually applied to practical use.^36^ CuO–Co_3_O_4_ cascade catalysts were investigated for NH_3_–SCO using *in situ* X-ray adsorption fine structure (XAFS) combined with a near ambient pressure (NAP) near edge X-ray absorption fine structure (NEXAFS). Bulk Co_3_O_4_ has an excellent ability to activate gaseous O_2_ on the surface, leading to outstanding NH_3_ conversion and N_2_O formation. The enhanced redox capacity of the CuO–Co_3_O_4_ interface leads to the efficient breakage of the N–O bond in N_2_O at a high temperature, forming N_2_ and reactive O. The reactive O will then react with NH_3_ to generate N_2_. We call this reaction pathway of decomposing N_2_O at high temperatures in NH_3_–SCO the decomposition of N_2_O (de-N_2_O) mechanism. The N_2_O formation rate and N_2_O dissociation rate are related to the concentration of surface Co_3_O_4_ and the CuO–Co_3_O_4_ interface, respectively. 90 wt % CuO–10 wt % Co_3_O_4_ not only has comparable catalytic performance to the Pt–Al_2_O_3_ catalyst but also can assist the Pt–Al_2_O_3_ catalyst in reducing N_2_O emissions from 20% to 3% at 723 K.

## 2 Experimental Section

### 2.1 Catalyst Preparation

The copper-based catalysts were prepared by coprecipitation using copper nitrate trihydrate (Cu(NO_3_)_2_·3H_2_O), cobalt nitrate hexahydrate (Co(NO_3_)_2_·6H_2_O), and ammonium hydroxide solution (28.0–30.0% NH_3_ basis) as starting materials. In a typical procedure, 2 g of nitrate precursor Cu(NO_3_)_2_·3H_2_O and the corresponding mass of Co(NO_3_)_2_·6H_2_O were dissolved in 15 mL of deionized water. Subsequently, the mixture was stirred for 10 min, and then NH_3_·H_2_O was added dropwise until the pH reached 9 under continuous stirring. After aging for 2 h, the sample was filtered and washed with deionized water, then dried at 60 °C overnight. The as-prepared sample was calcined at 550 °C in a muffle furnace for 4 h with a heating rate of 5 °C·min^–1^. Finally, the sample was slowly cooled to room temperature in the muffle furnace. The obtained solid was finely ground and sieved below 250 μm grain size.

By changing the loading of Cu, catalysts with different Cu aggregation states were prepared by the above method. For comparison, pure CuO and Co_3_O_4_ were also prepared using the above method.

The 1 wt % Pt–Al_2_O_3_ was prepared by the incipient wetness impregnation method. The H_2_PtCl_6_·(H_2_O)_6_ was dissolved in ethanol and then added into γ-Al_2_O_3_. γ-Al_2_O_3_ was purchased from Johnson Matthey. The sample was then dried at 60 °C overnight. Finally, the dried sample was calcined in air at 550 °C for 4 h at a heating rate of 5 °C min^–1^.

### 2.2 H_2_ Activation

The catalyst was placed in a fixed-bed flow reactor. At room temperature, 5% H_2_ balanced in He was introduced, and then the temperature was raised to 573 K with a total flow rate of 100 mL·min^–1^. After 30 min, 5% H_2_ balanced in He was switched to He, and the sample was slowly cooled to room temperature.

### 2.3 *Ex Situ* Characterizations

X-ray diffraction (XRD) measurements were performed on a StadiP diffractometer from STOE with a Mo source (Kα = 0.7093165 Å). The operating voltage and current are 40 kV and 30 mA, respectively. With a resolution of 0.015° for each step, the signals of 2θ in the range of 2°–40° were collected.

Scanning transmission electron microscope images (STEM) of the samples were obtained on a probe-corrected (CEOS) JEM ARM 200CF electron microscope (JEOL, Japan) at the electron Physical Science Imaging Centre (ePSIC). The sample was prepared by sprinkling dry catalyst powder on 400-mesh gold grids with a lacey carbon film. Using a 20 μm probe-forming aperture, the beam current was 33 pA with a 15.3 mrad probe convergence semiangle at 200 keV operation voltage. The samples were loaded onto Au grids by sprinkling a small amount of dry sample powder.

The energy dispersive X-ray spectrum (EDX) of the sample was obtained using the JEM ARM 200CF instrument equipped with a large solid angle dual EDX detector. The data were collected in STEM Illumination mode at 200 kV and corrected using special drift correction. Au TEM grids are used to avoid any Cu EDX signals from the grid. Each EDX spectrum image is 100 × 100 pixels in size, with 0.05 s exposure time per pixel.

Temperature-programmed desorption of N_2_O (N_2_O-TPD) was performed in a fixed-bed flow reactor. Prior to the experiment, the catalyst sample was pretreated in a He flow (50 mL·min^–1^) at 400 °C for 1 h. After this, the sample was cooled to 50 °C in He flow. The sample was exposed to 1% N_2_O (balance He) gas for 1 h at 50 °C, followed by flushing with He. After these treatments, N_2_O-TPD was carried out in a flow of He (80 mL·min^–1^) with a heating rate of 10 °C min^–1^.

X-ray absorption near edge structure (XANES) and extended X-ray absorption fine structure (EXAFS) of the Co K edge (7.709 keV) and Cu K edge (8.979 keV) were carried out in B18 in Diamond Light Source (UK) and BL01B1 in Spring8 (Japan). Samples with 1 wt % CuO loading were directly pressed into pellets for the fluorescence measurement of the Cu K edge. Other samples were diluted with boron nitride and pressed into a pellet with a diameter of 0.8 cm for the transmission measurement. CoO, Co_3_O_4_, CuO, and Cu_2_O standards were diluted with boron nitride and pressed into pellets for the transmission measurement. Co foil and Cu foil standards were used for energy shift calibration. For the EXAFS evaluation, at least three spectra were merged to improve the signal quality. XAFS data were processed using the Demeter software package (including Athena and Artemis).^37^ Athena software was used for the XANES analysis. Artemis software was used to fit the *k*^2^-weighted EXAFS data in real space with 3.0 Å^–1^ < *k* < 12.0 Å^–1^ and 1.0 Å < *R* < 3.3 Å. The calculated amplitude reduction factor S_0_^2^ from the EXAFS analysis of Cu foil was 0.878, which was used as a fixed parameter for EXAFS fitting. The calculated amplitude reduction factor *S*_0_^2^ from the EXAFS analysis of Co foil was 0.786, which was used as a fixed parameter for EXAFS fitting. The coordination number and bond length were calculated based on the reported structure from the Crystal open database: Cu (no. 9013014), CuO (no. 1011148), Co (no. 9011618), and Co_3_O_4_ (no. 1538531).

### 2.4 *In Situ* near-Edge X-ray Absorption Fine Structure (NEXAFS) Spectroscopy

*In situ* NEXAFS experiments for Co_3_O_4_ were performed at the ISISS beamline of BESSY II in Berlin (Germany). The X-ray is sourced from a bending magnet (D41) and a plane grating monochromator (PGM) with an energy range from 80 to 2000 eV (soft X-ray range) and a flux of 6 × 10^10^ photons s^–1^ with a 0.1 A ring current using a 111 μm slit and an 80 μm × 200 μm beam spot size. The reaction products were monitored online using an electron impact mass spectrometer (“PRISMA”, PFEIFFER VACUUM GmbH, Asslar, Germany)) connected directly to the main experimental chamber by a leak valve. The pressure in the specimen chamber was precisely controlled (UHV or 0.1–1 mbar) by simultaneous operation of several mass flow controllers for reactive gases and a PID-controlled throttle valve for pumping gas out. 100 mg samples of catalysts were pressed into pellets with a diameter of 6 mm. Sample pellets (6 mm diameter) were heated uniformly from an IR laser mounted on the rear part of the sample holder. Temperature control was realized by two K-type thermocouples. NEXAFS spectra at Co L edge (765–805 eV), O K edge (510–560 eV) and N K edge (390–420 eV) were measured in either the total electron yield (TEY) mode or the Auger electron yield (AEY) mode.

### 2.5 *In Situ* X-ray Absorption Fine Structure (XAFS) Experiments

*In situ* XAFS experiments for Co_3_O_4_ and 90 wt % CuO–Co_3_O_4_ (90CuCo) were performed at BL01B1 (Spring8, Japan). *In situ* XAFS of both catalysts was performed in a modified heating XAS cell (ASPF-20–03, Kyowa Vacuum). In the experiment, both catalysts were pressed into pellets with boron nitride (diameter 10 mm, thickness ca. 1 mm) and measured under various gas conditions at room temperature, 573 K, and 673 K. For Co_3_O_4_, around 5 mg of sample was mixed with 95 mg of boron nitride for measuring the Co K edge. For 90CuCo, around 5 mg of the sample was mixed with 95 mg boron nitride when measuring the Cu K edge, while 50 mg sample was mixed with boron nitride when measuring the Co K edge. The temperature was monitored by a K-type thermocouple and regulated by a PID controller. At 573 and 673 K, the catalysts were reduced by NH_3_ (5000 ppm of NH_3_ balanced in He) or NH_3_ + NO (5000 ppm of NH_3_ and 5000 ppm of NO balanced in He) and oxidized by NH_3_ + O_2_ (5000 ppm of NH_3_ and 5% O_2_ balanced in He), NH_3_ + NO + O_2_ (5000 ppm of NH_3_, 5000 ppm of NO, and 5% O_2_ balanced in He) or NO + O_2_ (5000 ppm of NO and 5% O_2_ balanced in He) with a total gas flow of 100 mL·min^–1^. The concentration of the respective gas was kept the same with balancing He while changing the composition of the gas flow. The outlet gases were sampled continuously with a quadrupole mass (Q-Mass) spectrometer. XANES spectra of each gas composition were recorded between 7400 and 8910 eV for the Co K edge and 8660–10170 eV for the Cu K edge in transmission mode with Si(111) crystal monochromator. The spectra processing and linear combination fitting (LCF) analysis were also processed with Athena, and the EXAFS fitting was processed using Artemis.^37^

### 2.6 *In Situ* Diffusion Reflection Infrared Fourier Transform Spectroscopy (DRIFTS)

*In situ* diffusion reflection infrared fourier transform spectroscopy (DRIFTS) was carried out on a Thermo Scientific NiCOLET iS50 spectrometer. Before testing, the powder sample was placed in the sample chamber. The detailed test programs could be divided into three parts as follows:

(1) The sample was heated under Ar flow from room temperature (RT) to 400 °C with a ramping rate of 20 °C·min^–1^. The background profiles were collected at RT, 200 °C, 300 °C, and 400 °C, respectively. After that, the sample was cooled under a pure O_2_ flow.
(2) At RT, the gas flow (5% NH_3_, 50% O_2_, 45% Ar, 50 mL·min^–1^) was introduced into the reaction cell. Under this predetermined atmosphere, the sample was heated from RT to 400 °C (20 °C min^–1^). During the heating process, the *in situ* DRIFTS spectra were detected at RT, 200 °C, 300 °C, and 400 °C, respectively.
(3) At 400 °C, the *in situ* DRIFTS spectra were collected with a consecutive switch of predetermined gases and Ar. Followed by program (2), Ar flow was used to purge the sample cell for 5 min. After that, a mixed gas (100 mL·min^–1^) of 2.5% NH_3_, 2.5% N_2_O, 25% O_2_, and 70% Ar was switched to the sample room, and the signal was detected. After the purge of Ar, another mixed gas (3.3% N_2_O, 33.3% O_2_% and 63.4% Ar) with a flow rate of 75 mL·min^–1^ was introduced, followed by signal collection. Then, a switch of Ar, 5% N_2_O/Ar (50 mL·min^–1^), Ar, and mixed gas (3.3% NH_3_, 3.3% N_2_O, and 93.4% Ar, 75 mL·min^–1^) was carried out. Under the predetermined reaction gases, the *in situ* DRIFTS spectra were recorded .

### 2.7 Catalytic Performance Measurement

The selective catalytic oxidation of ammonia was evaluated in a fixed-bed flow system using a stainless-steel flow reactor (i.d. = 9.75 mm, 300 mm length). The composition and flow rate of the inlet gas mixture were set by the mass flow controller. A typical reaction gas composition was 5000 ppm of NH_3_, 5 vol % O_2_, and balance He. The flow rate of the mixed gas was 100 mL·min^–1^. Typically, 50 mg of catalyst powder diluted with 100 mg SiC (400 mesh) was placed in the reaction tube, and the product is detected with the quadrupole mass spectrometer (MS) quantitative gas analyzer (Hiden Analytical, UK). The Hiden QGA can be used to determine how overlapping species may be separated using soft ionization techniques, which allows one to selectively ionize different gases by setting the ionization energy for a particular mass. The reaction was studied in the temperature range of 423–723 K. After reaching a steady state at each reaction temperature, the reaction was maintained for at least 30 min to measure the MS signals of reactants (NH_3_ and O_2_) and products (N_2_, N_2_O, NO, and NO_2_).

### 2.8 DFT Calculations

The DFT+U calculations were utilized through the calculation software QuantumATK.^38^ The exchange-correction interactions were described by the projector augmented wave (PAW) pseudopotentials with the Perdew–Burke–Ernzerhof (PBE) functional.^39^ The values of U were carefully chosen according to the relevant paper, which is 4 eV for Co atoms.^40^ The Brillouin zone was sampled using a 9 × 9 × 9 Monkhorst–Pack k-point mesh and 520 eV cutoff energy for the primitive cell lattice optimization, while 1 × 1 × 1 k-points are employed for subsequent adsorption calculations. During the optimization, the convergence criteria were set to 0.03 eV/A and 10^–5^ eV for force and energy, respectively. We also chose the (100) surfaces of Co_3_O_4_ owing to their lower surface energy and greater stability.^41^

The adsorption energy per molecule was calculated as follows:^42^
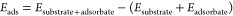
in which *E*_substrate+adsorbate_ is the total energy of the whole system, which includes the absorbate and the substrate structure. *E*_substrate_ and *E*_adsorbate_ are the energies of substrate structure and adsorbed gas molecule, respectively. According to the equation, the negative adsorption energy indicates the existence of adsorption, while the positive one means no evident adsorption interactions.

## 3 Results and Discussion

**Figure 1:**
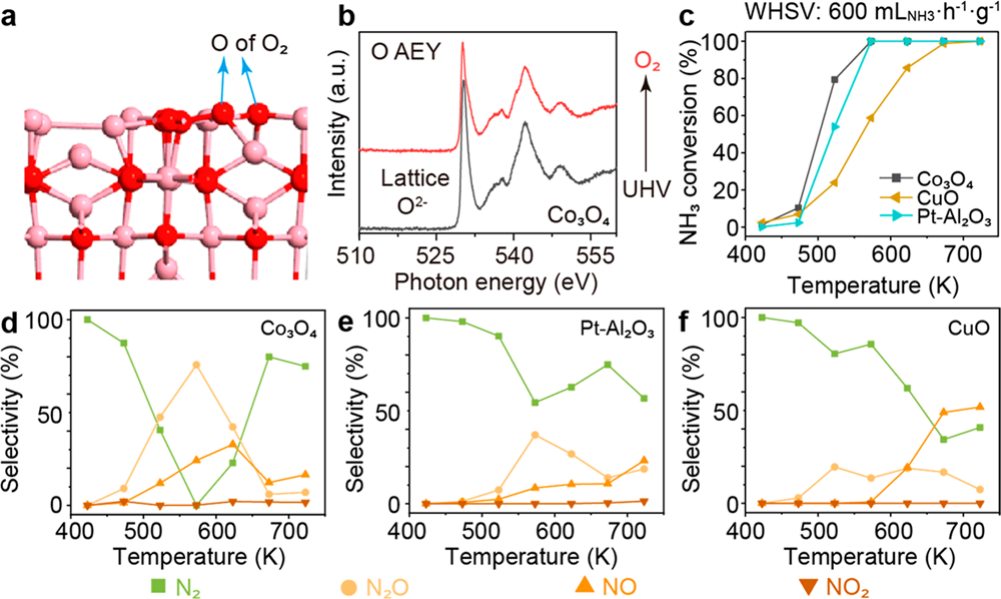
Co_3_O_4_, CuO and 1 wt % Pt/Al_2_O_3_ catalysts. (a) DFT calculation of O_2_ over Co_3_O_4_ (100), see Figure S4 for details. The DFT calculations of NO and NH_3_ adsorption are shown in Figures S5 and S6. (b) O K edge NEXAFS spectra of Co_3_O_4_ under UHV and 0.3 mbar of O_2_ at 298 K. (c) NH_3_ conversion as the function of temperature for Co_3_O_4_, Pt/Al_2_O_3_, and CuO catalysts. (d–f) Selectivity of N_2_, N_2_O, NO, and NO_2_ as the function of temperature for (d) Co_3_O_4_, (e) Pt/Al_2_O_3_, and (f) CuO catalysts. Reaction conditions are as follows: *m*_cat_ = 50 mg, 5000 ppm of NH_3_, 5% O_2_ balanced in He, gas flow of 100 mL·min^–1^, weight hourly space velocity (WHSV) = 600 mL_NH3_·h^–1^·g^–1^.

Co_3_O_4_, which is composed of mixed valence Co^2+^ and Co^3+^ with a spinel structure, has been extensively studied for the oxidation of CO and the combustion of CH_4_.^43,44^ We hypothesize that the presence of Co(II) and its redox with Co(III) can activate gaseous O_2_, which is the main reason for the fast oxidation of NH_3_. Density functional theory (DFT) calculations support this hypothesis, showing the weakened O–O bond and the increased bond length of the oxygen molecule on the Co_3_O_4_ (100) surface (Figure 1a) with an adsorption energy of −5.27 eV (Table S1). In comparison, the calculated results over the CuO (001) surface show physical O_2_ adsorption with −0.13 eV adsorption energy.^45^

Such an O_2_ activation is observed in the O K edge near edge X-ray absorption fine structure (NEXAFS). After oxygen was introduced, the peak of gaseous oxygen in the O K edge (Figure S1, 530.6 eV) was not observed on the surface of Co_3_O_4_, while a peak at 530.1 eV was observed (Figure 1b). Compared with ultrahigh vacuum (UHV) and NH_3_ conditions, this peak only appears under O_2_ conditions (Figures S2 and S3). Such a peak shift (0.5 eV) toward low photon energy indicates the formation of more reactive O_2_^δ-^ species on the surface, in which the 2pπ* peak shifts to lower energy.^46,47^ Surface superoxo- or peroxo-like O_2_ species are active in the oxidation of CO^48^ and hydrocarbons^49^ (e.g., toluene), and such reactive oxygen species are usually formed by O_2_ molecules on O vacancies^50^ on the surface of transition metal oxides.^51,52^ For Co_3_O_4_, the O vacancy near the Co^2+^ species can activate O_2_, taking electrons from Co^2+^ to form O_2_^δ-^, whereas Co^2+^ is oxidized to Co^3+^.^53^ The NH_3_–O_2_^δ-^ complex has been reported as a key intermediate in the oxidation of NH_3_.^54^ The catalytic activity of NH_3_–SCO at low temperatures was also found to scale proportionally with the concentration of Cu(II) superoxo species.^55^ Both Co_3_O_4_ and CuO catalysts prepared by precipitation were evaluated for NH_3_ oxidation in the temperature window of 423–723 K (Figure 1c–f) and compared with 1 wt % Pt–Al_2_O_3_. Co_3_O_4_ is a very active material for oxidation reactions and is more active than Pt–Al_2_O_3_ in NH_3_ oxidation, with 100% conversion at 573 K (Figure 1c). Both Co_3_O_4_ and Pt–Al_2_O_3_ show a seagull shape of selectivity trend, with high N_2_ selectivity below 500 K and above 623 K, which is less discussed in the literature. In the mid-temperature range between 500 and 623 K, the major byproduct is N_2_O (Figure 1d and e). In comparison, CuO can still maintain a high N_2_ selectivity in this region (Figure 1f), though with a lower conversion. These results suggest that O_2_^δ-^ on the surface of Co_3_O_4_ is the active site for NH_3_ conversion and N_2_O formation.

To understand the observed catalytic trends for Co_3_O_4_ catalysts, we considered the imide mechanism of low-temperature N_2_O formation.^56,57^ Adsorbed NH_3_ would first react with the active oxygen species to form NH_x_* (eqs 1 and 2). The NH* is subsequently oxidized to form the nitroxyl species (HNO*) (eq 3). N_2_ can be formed in the reaction between NH* and HNO* (eq 4). N_2_O is commonly accepted to be formed in the reaction of HNO* (eq 5). The product distribution is therefore determined by the relative concentration of NH_x_* and HNO* species on the catalyst surface. At low temperatures, the HNO* density on the surface is relatively low, leading to high selectivity toward N_2_. Increasing the temperature, more NH_3_ is oxidized to HNO*, resulting in N_2_O formation. This explains the trend of N_2_ toward N_2_O between 400 and 573 K.
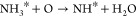
1
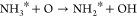
2
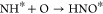
3
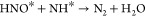
4
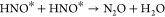
5

However, the imide mechanism cannot explain the shift from N_2_O to N_2_ above 573 K. Here, we propose a new mechanism for NH_3_–SCO, which is the decomposition of N_2_O (de-N_2_O) with the help of NH_3_ to form N_2_. In this mechanism, N_2_O first dissociates to N_2_ and O* (eq 6). After that, O* can react with absorbed NH_x_* to form N_2_ (eq 7). The breaking of N–O bonds in N_2_O is usually carried out at high temperatures. The removal of O* by NH_3_ can further promote the dissociation of N_2_O. As a result, the selectivity changes from N_2_O to N_2_ at high temperatures.
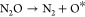
6
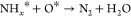
7

Co_3_O_4_ generates N_2_O between 500 and 623 K, whereas CuO maintains high N_2_ selectivity. We hypothesize that the addition of CuO to Co_3_O_4_ will convert the newly formed N_2_O into N_2_ following eqs 6 and 7.

To prove this, we prepared a range of CuO–Co_3_O_4_ catalysts via the coprecipitation method with 1, 25, 90, and 99 wt % CuO loadings (denoted as 1CuCo, 25CuCo, 90CuCo and 99CuCo, respectively). Co_3_O_4_ nanoparticles are obtained by the precipitation method with an average size of 50 nm (Figure S7). Coprecipitating with 1 wt % Cu does not change the morphology of the catalysts (Figure S8). Co_3_O_4_(111) facets are observed in the bright field-scanning transmission electron microscopy images (BF-STEM) for 1CuCo (Figure S9a). The presence of Cu in 1CuCo is further confirmed in the energy dispersive X-ray spectrum (EDX), showing Cu Kα emission at 8.05 keV (Figure S10). Cu species are uniformly dispersed on Co_3_O_4_, as shown in the homogeneous Cu map compared to that of Co (Figure S9b). XRD patterns further confirm the crystallinity of CuO and Co_3_O_4_ (Figure S11). The diffraction features of CuO can be observed with CuO loadings above 25 wt %, and the diffraction characteristics of Co_3_O_4_ are relatively weak when the CuO loading is 90%.

The oxidation states and coordination environments for 1CuCo, 25CuCo, and 90CuCo catalysts were examined by X-ray absorption near edge spectroscopy (XANES) and extended X-ray absorption fine structure (EXAFS). Cu in these three catalysts has a similar peak shape and white line position to those of the CuO standard, showing the Cu(II) configuration. The obvious separation between 1s → 4p_*z*_ and 1s → 4p_*xy*_ is observed in 25CuCo and 90CuCo, which is due to the elongated octahedral coordination of Cu in CuO.^58,59^ In comparison, XANES spectra show no obvious separation between 1s → 4p_*z*_ and 1s → 4p_*xy*_ for 1CuCo (Figure S9c,d), suggesting a reduced Jahn–Teller effect. We speculate that Cu(II) replaces the position of tetrahedral-coordinated Co(II) in Co_3_O_4_ in 1CuCo, thus showing similar XANES spectra compared to CuCo_2_O_4_.^60^ For 25CuCo, the coordination number of Cu–Cu (1) is similar to that of the CuO standard, while the Cu–Cu (2) scattering is much lower than that of 90CuCo and the CuO standard (Figure S9e, fitting results in Figure S12 and Table S2), indicating the formation of a small CuO cluster that lacks long-range Cu–Cu order. Co in these three catalysts is in the form of Co_3_O_4_, as shown in the Co K edge XANES spectra (Figure S9g and h). The coordination number of Co–Co (2) of 90CuCo is less than that of Co_3_O_4_ (Figure S9f, fitting results in Figure S13 and Table S3) and is consistent with the XRD results, showing that Co_3_O_4_ clusters are formed over CuO. Therefore, highly dispersed Cu over Co_3_O_4_ (1CuCo), CuO cluster over Co_3_O_4_ (25CuCo), and Co_3_O_4_ cluster over CuO (90CuCo) were synthesized by changing the Cu loading.

**Figure 2:**
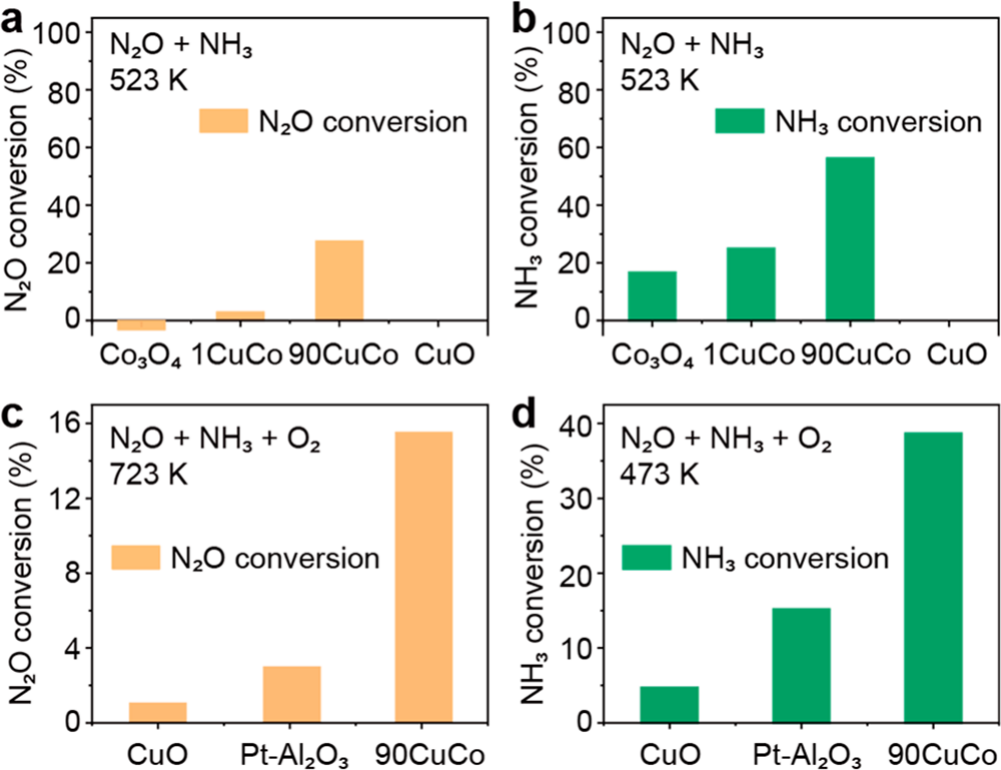
The catalytic performance in N_2_O reduction by NH_3_. (a) Conversion of N_2_O for CuO–Co_3_O_4_ catalysts at 523 K. (b) Conversion of NH_3_ for CuO–Co_3_O_4_ catalysts at 523 K. Reaction conditions are as follows: 120 mg of catalyst, 5000 ppm of NH_3_, 5000 ppm of N_2_O balanced in He, and gas flow of 80 mL·min^–1^. (c) Conversion of N_2_O for pure CuO, 1 wt % Pt–Al_2_O_3_, and 90 wt % CuO–Co_3_O_4_ catalysts at 723 K. (d) Conversion of NH_3_ for pure CuO, 1 wt % Pt–Al_2_O_3_, and 90 wt % CuO–Co_3_O_4_ catalysts at 473 K. Reaction conditions are as follows: 120 mg of catalyst, 3000 ppm of NH_3_, 4500 ppm of N_2_O, 5% O_2_ balanced in He, and gas flow of 100 mL·min^–1^. Negative N_2_O conversion suggested the formation of N_2_O.

We then evaluated the reduction of N_2_O by NH_3_ for the CuO–Co_3_O_4_ catalysts. CuO cannot convert N_2_O to N_2_ below 723 K. Co_3_O_4_ can convert N_2_O to N_2_, but only at 623 K or above (Figure S14). This also agrees with the trend of N_2_O selectivity in NH_3_ oxidation (Figure 1d). The addition of CuO to Co_3_O_4_ created two metal surfaces for the reaction. With 1 wt % CuO on Co_3_O_4_ (1CuCo), the N_2_O conversion temperature is reduced by 50 K. The 90 wt % CuO with Co_3_O_4_ (90CuCo) further reduced the required temperature by another 50 K, so that the N_2_O conversion can be achieved from 500 K onward. At 523 K, the N_2_O conversion at 90CuCo is nine-times higher than that of 1CuCo (Figure 2a), with the highest NH_3_ conversion (Figure 2b).

The N_2_O decomposition reaction was reported to be notably hindered by the presence of O_2_.^12^ Introducing O_2_, 90CuCo can still effectively convert N_2_O at 723 K (Figure S15), showing 5× and 14 × N_2_O conversion than 1 wt % Pt/Al_2_O_3_ and CuO (Figure 2c), respectively. With the promotion of N_2_O, 90CuCo has higher NH_3_ conversion than 1 wt % Pt/Al_2_O_3_ (Figure 2d and Figure S15). Without NH_3_, 90CuCo has lower N_2_O conversion than Co_3_O_4_ and 1CuCo (Figure S16), suggesting the important role of NH_3_ in the decomposition of N_2_O. To further derive the important role of NH_3_ in N_2_O decomposition, we compared N_2_O conversion for 90CuCo with the presence and absence of NH_3_. To prevent NH_3_ from being oxidized to N_2_O, we did not introduce O_2_. We found that NH_3_ is essential for the conversion of N_2_O at low temperatures (below 723 K). In the absence of NH_3_, N_2_O is essentially undecomposable at low temperatures (Figure S17). In contrast, more than 50% of N_2_O decomposes at 573 K in the presence of NH_3_.

The introduction of NH_3_ can significantly facilitate the decomposition of N_2_O in the absence of O_2_ (NH_3_ + N_2_O conditions). At the same time, active O formed from N_2_O decomposition also contributes to the consumption of NH_3_. 90CuCo exhibits worse NH_3_ oxidation activity than Pt/Al_2_O_3_ without the introduction of N_2_O (NH_3_ + O_2_ conditions) at 523 K. After the introduction of N_2_O (NH_3_ + N_2_O + O_2_ conditions), 90CuCo obtained a higher NH_3_ conversion than Pt/Al_2_O_3_ (Figure S15). This shows that N_2_O significantly facilitates the conversion of NH_3_ for the 90CuCo catalyst, which reflects the reaction between NH_3_ and N_2_O. The possible reason for this is that the reactive O from N_2_O decomposition contributes to the conversion of NH_3_, whereas Pt/Al_2_O_3_ is less capable of N_2_O decomposition. The results show that 90CuCo is a very active catalyst for the breakage of the N–O bond of N_2_O with the help of NH_3_, which will be promising in eliminating both NH_3_ and N_2_O emissions.

**Figure 3:**
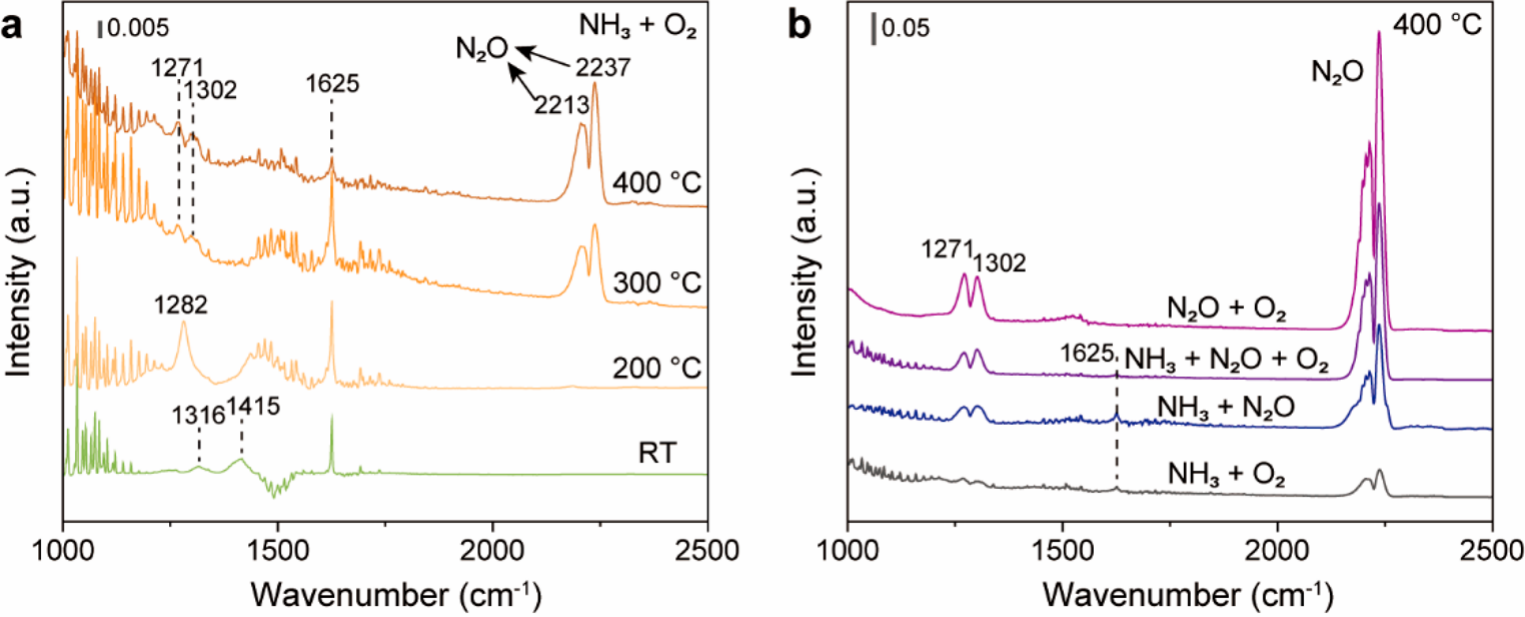
*In situ* DRIFTS investigations. DRIFT spectra of adsorbed species on the 90CuCo under (a) NH_3_ + O_2_ from room temperature to 400 °C and (b) different gas conditions (NH_3_ + O_2_, NH_3_ + N_2_O, NH_3_ + N_2_O + O_2_, and N_2_O + O_2_) at 400 °C.

To validate that the decrease in N_2_O emissions resulted from its further decomposition, we performed an *in situ* DRIFTS experiment from room temperature to 400 °C under NH_3_ + O_2_ (Figure 3a). Strong bands at 1625 cm^–1^ are observed for 90CuCo from room temperature to 400 °C under NH_3_ + O_2_, which are assigned to the asymmetric bending vibrations of coordinated NH_3_.^61^ The bands at 1316 and 1415 cm^–1^ under room temperature can be ascribed to NH_3_ adsorbed on Lewis acid sites^21^ and the asymmetric bending vibrations of N–H bonds^62^ in NH_4_^+^, respectively. The band at 1282 cm^–1^ observed at 200 °C was attributed to monodentate nitrates.^63^ No adsorption signals for N_2_O were observed at both room temperature and 200 °C. Increasing the temperature to 300 °C, four adsorption peaks can be observed at 2237, 2213, 1302, and 1271 cm^–1^ in Figure 3, which were confirmed to be the stretching frequencies of N–N and N–O bonds in N_2_O.^64^ At 400 °C, the N_2_O signal can still be observed, suggesting that large amounts of N_2_O can still be produced and adsorbed on the catalyst surface.

For 90CuCo, the formation of monodentate nitrates at 200 °C suggests that N_2_ formation under low temperatures for 90CuCo follows the i-SCR mechanism. However, the nitrate species are less detectable under high temperatures (Figure 3a), indicating a different mechanism for N_2_ formation (see the detailed discussion in Text S1). Although there are other possible reaction paths to generate N_2_, we consider the decomposition of N_2_O to N_2_ to be the main reaction path at high temperatures.

To confirm the role of NH_3_ and O_2_ for the decomposition of N_2_O, we performed *in situ* DRFITS at 400 °C under different gas conditions (NH_3_ + O_2_, NH_3_ + N_2_O, NH_3_ + N_2_O + O_2_, and N_2_O + O_2_, Figure 3b). The intensity of the N_2_O bands of NH_3_ + N_2_O + O_2_ was stronger than that of N_2_O + NH_3_. These results suggest that O_2_ inhibits the reduction of N_2_O by NH_3_. The possible reasons are (1) O_2_ can oxidize NH_3_ to N_2_O and therefore NH_3_ cannot be a selective reductant and (2) the competitive adsorption of O_2_ into the active sites. We found that the intensity of the N_2_O band of NH_3_ + N_2_O + O_2_ was significantly weaker than that of N_2_O + O_2_ (Figure 3b). The weaker intensity of the N_2_O signals could potentially be attributed to various factors, including competitive adsorption. However, NH_3_ introduced at high temperatures can also react with oxygen to form additional N_2_O on the surface. These results reflect the importance of NH_3_ for reducing the surface coverage of N_2_O. *In situ* DRIFTS further supports our speculation that NH_3_ is oxidized to N_2_O at high temperatures (above 300 °C) and the as-formed N_2_O is decomposed to produce N_2_.

**Figure 4:**
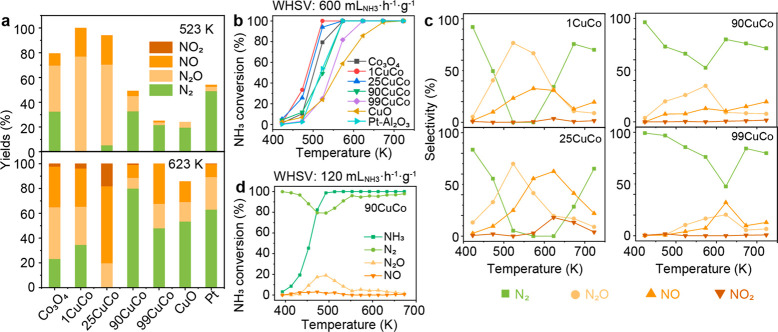
Catalytic performance in NH_**3**_-SCO for CuO–Co_3_O_4_. (a) Yields of N_2_, N_2_O, NO, and NO_2_ at 523 and 623 K. (b) NH_3_ conversion as a function of temperature. (c) Selectivity of N_2_, N_2_O, NO, and NO_2_ as the function of temperature for CuO–Co_3_O_4_ catalysts. (d) NH_3_ conversion and product selectivity as the function of temperature for the 90 wt % CuO–Co_3_O_4_ catalyst. Reaction conditions are as follows: (a–c) *T*_bed_ = 423–723 K, *m*_cat_ = 50 mg, 5000 ppm of NH_3_, 5% O_2_ balanced in He, gas flow of 100 mL·min^–1^, WHSV = 600 mL_NH3_·h^–1^·g^–1^; (d) T_bed_ = 393–773 K, m_cat_ = 50 mg, 1000 ppm of NH_3_, 5% O_2_ balanced in He, gas flow of 100 mL·min^–1^, and WHSV = 120 mL_NH3_·h^–1^·g^–1^.

The CuO–Co_3_O_4_ catalysts were then evaluated for NH_3_ oxidation in the temperature window of 423–723 K (Figure 4) and compared with 1 wt % Pt–Al_2_O_3_. 1CuCo has a reactivity profile similar to that of Co_3_O_4_ (Figure 4a). With the increase of the Cu loading, the NH_3_ conversion decreases due to the reduced amount of Co on the surface. 90CuCo has a slightly lower NH_3_ conversion rate than Pt/Al_2_O_3_ (Figure 4b) but higher N_2_ selectivity at 723 K (71% vs 57%, Figure 4c).

Under realistic NH_3_ slip conditions (1000 ppm of NH_3_), NH_3_ can be completely converted by 90CuCo at 493 K (Figure 4d). More importantly, 90CuCo has higher N_2_ selectivity and similar selectivity trends under a low NH_3_ concentration, providing a wide operation window from 553 to 773 K, with the N_2_ yield exceeding 90% (Table S4). Such a wide operation window exceeds those of most catalysts reported in the literature (Figure S18). 90CuCo shows excellent catalytic stability without any noticeable drop during the 100 h reaction (Figure S19). This suggests the potential of replacing expensive Pt in the industrial NH_3_ slip process. In order to evaluate the performance of the catalyst under more realistic NH_3_ slip conditions, we conducted measurements on the 90CuCo catalyst at a low NH_3_ concentration (1000 ppm) with a significant amount of H_2_O (5%). It was observed that, in the presence of 5% H_2_O, the activity of the 90CuCo catalyst experienced a slight decrease while maintaining a selectivity of approximately 90% at elevated temperatures (Figure S20). This shows the potential of 90CuCo in practical applications.

With 1 wt % Co_3_O_4_ loaded on CuO, the activity of 99CuCo is slightly higher than that of pure CuO, suggesting the importance of Co_3_O_4_ in O_2_ activation at low temperatures. We have successfully tested our hypothesis that the bifunctional Co_3_O_4_ and CuO surface with the desired ratio at 90CuCo converts the as-formed N_2_O into N_2_ throughout the testing temperatures without losing too much NH_3_ oxidation activity.

**Figure 5:**
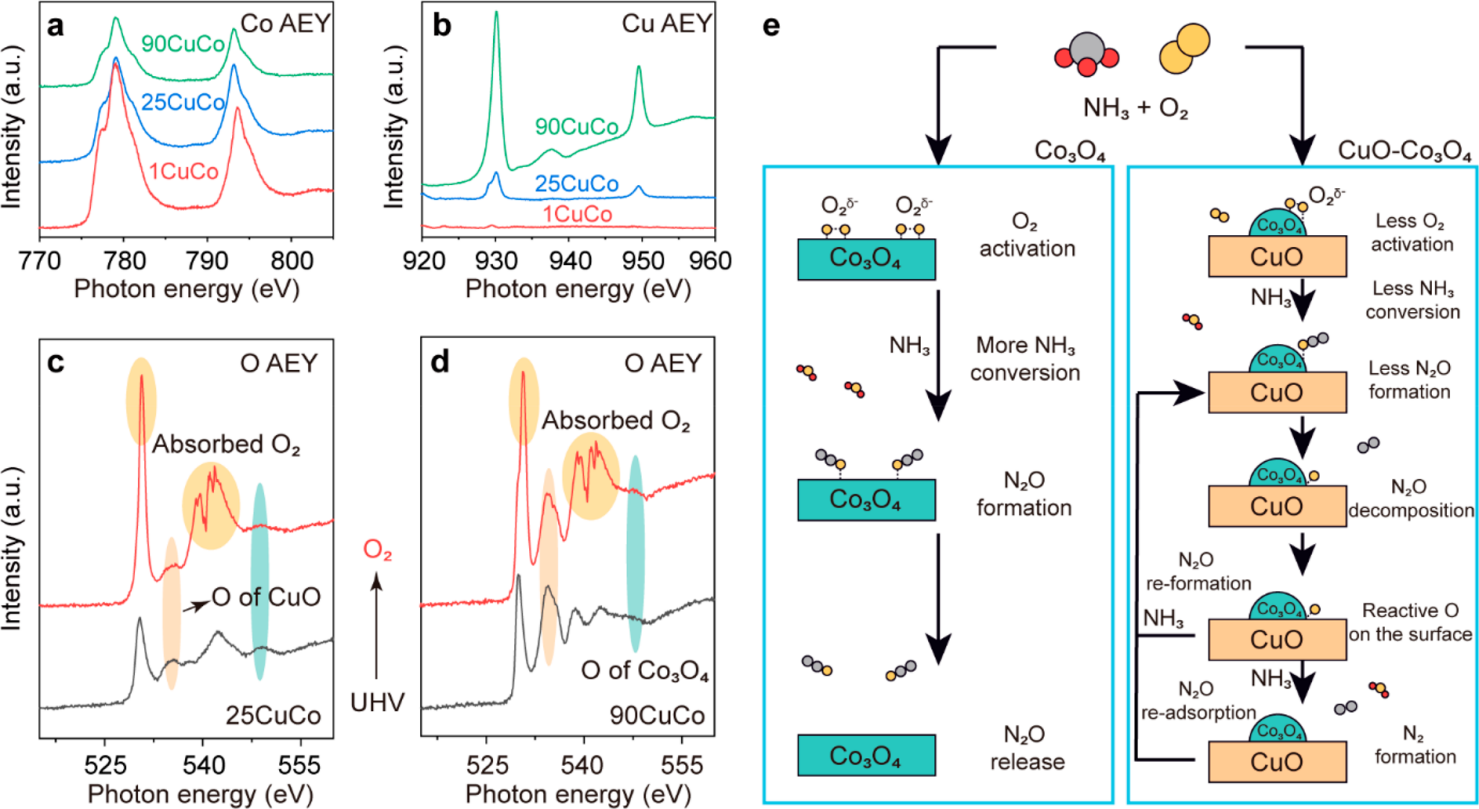
NEXAFS study of CuO–Co_3_O_4_ catalysts. (a) Co L edge NEXAFS spectra of CuO–Co_3_O_4_ catalysts under UHV at 298 K. (b) Cu L edge NEXAFS spectra of CuO–Co_3_O_4_ catalysts under UHV at 298 K. (c) the O K edge NEXAFS spectra of 25 wt % CuO–Co_3_O_4_ under UHV or O_2_ (0.3 mbar) at 298 K. (d) O K edge NEXAFS spectra of 90 wt %CuO–Co_3_O_4_ under UHV or O_2_ (0.3 mbar) at 298 K. (e) Simplified reaction pathways for cobalt-catalyzed selective oxidation of ammonia.

NAP-NEXAFS experiments were carried out to provide surface chemical details of 1CuCo, 25CuCo, and 90CuCo. 1CuCo has the highest amount of Co species on the surface with a weak Cu signal. 25CuCo has a higher Cu feature than 1CuCo, confirming the presence of CuO clusters on the surface. 90CuCo shows the majority of CuO on the surface (Figure 5a and b). With different surface metal ratios, lattice O of CuO and Co_3_O_4_ with different intensities can be detected over 25CuCo and 90CuCo. Unlike the pure Co_3_O_4_ (Figure 1b), surface physically adsorbed O_2_ can be observed over 25CuCo and 90CuCo (Figure 5c and d), showing a weaker activation of the O_2_, which leads to decreased NH_3_ conversion for 90CuCo (Figure 4b). For Co_3_O_4_, active surface O species lead to the rapid formation of N_2_O, which directly desorbs at low temperatures. For CuO–Co_3_O_4_, the as-formed N_2_O tends to decompose to N_2_ and O* on the surface. O* can then be removed by unreacted NH_3_, forming N_2_. After the surface of O* is scavenged, N_2_O can continue to decompose on the surface again (Figure 5e).

To understand the transition of products from N_2_O to N_2_ at high temperatures for Co_3_O_4_, the Co L edge and the O K edge were studied under different NH_3_/O_2_ ratios. The adsorbed active molecular oxygen can provide oxygen for the oxidation of NH_3_, but the substrate lattice can also do this. In the latter case, the task of gas-phase O_2_ is to restore the catalyst by refilling the oxygen vacancies. This is a so-called Mars–van-Krevelen (MvK) mechanism, which has been reported as a method of CO oxidation on Co_3_O_4_.^44,65^ In addition to oxygen, N_2_O can also be considered as an O-donor molecule because of its low N_2_-oxygen affinity of 40 kcal/mol.^66^ Therefore, surface oxygen vacancies have been discussed as active sites for the decomposition of N_2_O,^67^ increasing the chemisorption of N_2_O on the catalyst surface.^68^ To examine the surface O vacancies during the reaction, the NH_3_ and O_2_ mixtures at different ratios are introduced to Co_3_O_4_ at 573 and 673 K (for the spectra and the corresponding mass spectrometry (MS) signals, see Figures S21–S23). Co(III) to Co(II) transition starts under NH_3_/O_2_ = 1:9 at 573 K, indicating the formation of stable surface O vacancies at the Co_3_O_4_ surface under low temperature despite the excess of oxygen. Changing from NH_3_ to O_2_, Co(II) can be oxidized to Co(III) at NH_3_/O_2_ = 1:1 at 673 K, suggesting that O vacancies are more likely to be refilled at high temperatures. This explains that N_2_O starts to decompose only on the surface of Co_3_O_4_ at high temperatures. CuO–Co_3_O_4_ catalysts were found to be more active for N_2_O decomposition (Figure 2a). Hence, the promotion of CuO in CuO–Co_3_O_4_ catalysts was followed by *in situ* XAFS investigations.

**Figure 6:**
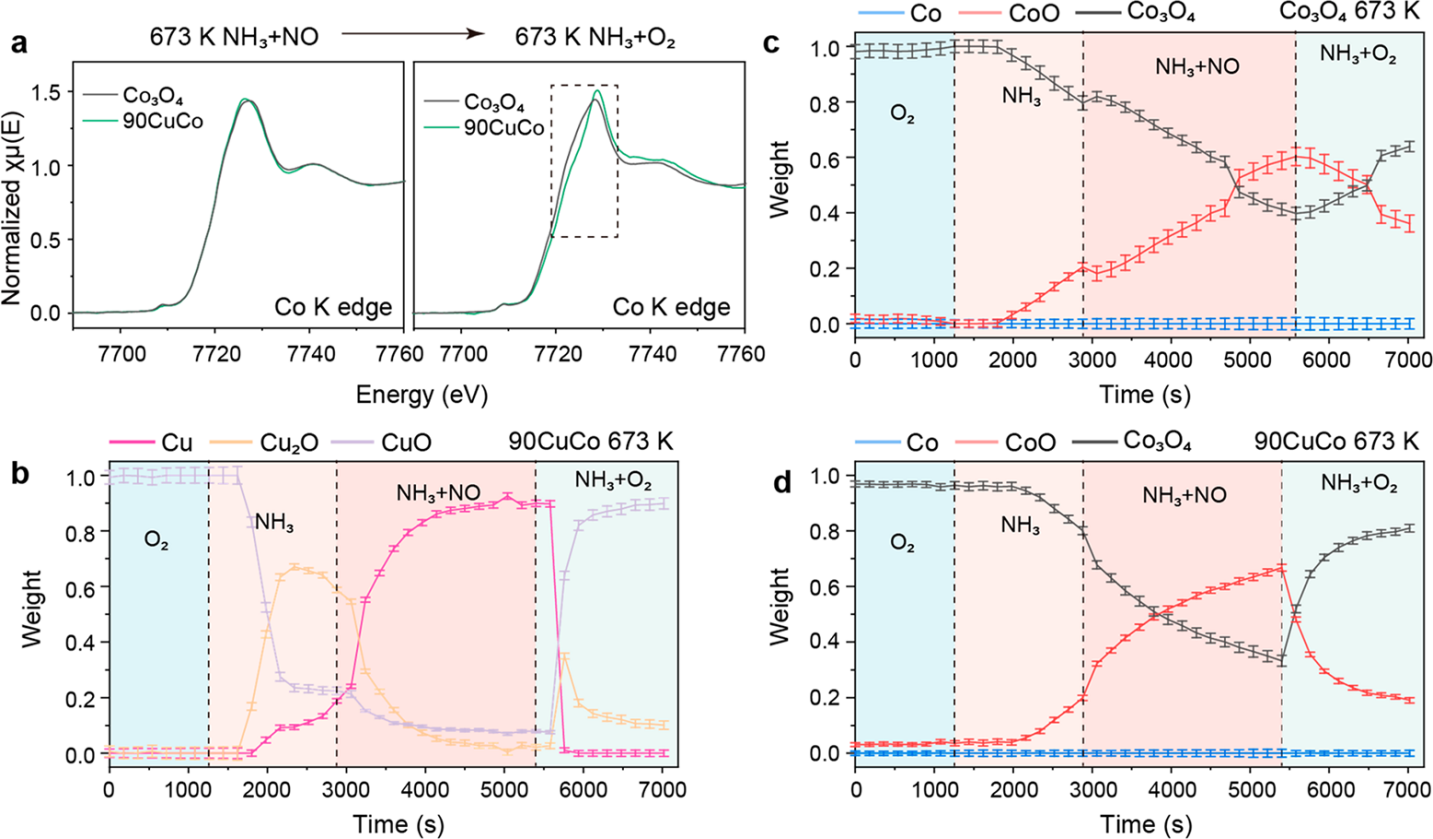
*In situ* XAFS study of Co_3_O_4_ and 90CuCo. (a) Co K edge XANES spectra of Co_3_O_4_ and 90CuCo under NH_3_ + NO and NH_3_ + O_2_ conditions at 673 K. (b) Time-resolved Cu profile when changing from oxidative to reductive conditions and back to oxidative conditions for 90CuCo at 673 K. (c) Time-resolved Co profile when changing from oxidative to reductive conditions and back to oxidative conditions for Co_3_O_4_ at 673 K. (d) Time-resolved Co profile when changing from oxidative to reductive conditions and back to oxidative conditions for 90CuCo at 673 K.

The cleavage of the N–O bond of N_2_O requires the catalyst to directly extract the O in the N_2_O molecule, which means that the redox of the catalyst is involved. Electron donation from Co(II) to N_2_O results in the formation of Co(III) species, while Co(III) can be reduced back to Co(II) by NH_3_. Pure CuO cannot effectively decompose N_2_O, but the addition of CuO into Co_3_O_4_ significantly increases the N_2_O decomposition ability. Therefore, both the redox of Co and Cu are crucial for the removal of N_2_O, which were studied by *in situ* XAFS at Co K and Cu K edges of Co_3_O_4_ and 90CuCo (for the spectra, see Figures S24–S29). The linear combination fitting (LCF) is used to quantify the oxidized and reduced Co/Cu species. At 573 K, bulk CuO can be partially reduced under NH_3_ (Figure S28), while bulk Co_3_O_4_ in Co_3_O_4_ and 90CuCo cannot be reduced (Figures S24 and S25), indicating the better reducibility of CuO. Increasing temperature to 673 K, the Cu shows a dramatic reduction to Cu_2_O from O_2_ to NH_3_ (Figure S29), whereas 20% of Co_3_O_4_ is reduced to CoO (Figure S27). Further reduction is observed under NO + NH_3_ conditions, with metallic Cu and more CoO formation. Co in bulk Co_3_O_4_ has similar reduction behavior compared to 90CuCo. Changing from reduction conditions to NH_3_ + O_2_ conditions, both Co and Cu are in the oxidized form at 573 K and are only slightly reduced at 673 K, while the Co(II) in Co_3_O_4_ remains stable (Figure 6a). The change of oxidation states of Cu consists well with the change of oxidation states of Co, suggesting the formation of Co(II)–Vo–Cu(I), which can dissociate the N–O bond (eq 8).
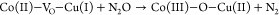
8
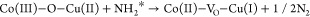
9

The Co and Cu reduction/oxidation rates can be determined with time-resolved XAFS. The samples were diluted with boron nitride to ensure that the amount of Co was similar for both samples when measuring the Co K edge. The spectra were recorded every 150 s while the catalytic activity was simultaneously monitored by an online mass spectrometer connected to the outlet of *in situ* tube (Figures S30 and S31). The LCF analysis of Co K edge XANES under reduction conditions shows a trend similar to those of Co_3_O_4_ and 90CuCo samples regarding the fast reduction of CuO (Figure 6b). In the oxidation process, only 4% CoO is oxidized to Co_3_O_4_ for pure Co_3_O_4_ in 300 s (Figure 6c). In comparison, the Co in 90CuCo has a faster oxidation rate, with oxidation of 31% CoO to Co_3_O_4_ in the same time (Figure 6d). The higher Cu reduction/oxidation rate suggests that Cu acts as the catalyst or promoter for Co(II) oxidation, which is then important for N_2_O removal. As a result, the interface of CuO–Co_3_O_4_ is the active site for the dissociation of N_2_O (eq 8 and 9).

To summarize the *in situ* studies and reaction profiles, the activation of gaseous O_2_ over the Co_3_O_4_ surface is the key to the NH_3_ conversion and N_2_O formation. The interface of CuO–Co_3_O_4_ has an enhanced redox ability, promoting the dissociation of N_2_O at high temperatures. The reactive O produced by N_2_O cleavage in turn contributes to the oxidation of NH_3_. Controlling the ratio of CuO and Co_3_O_4_ on the surface can modulate the rates of N_2_O formation and N_2_O removal, thus regulating product selectivity. In this case, 90CuCo is found to be optimal.

**Figure 7:**
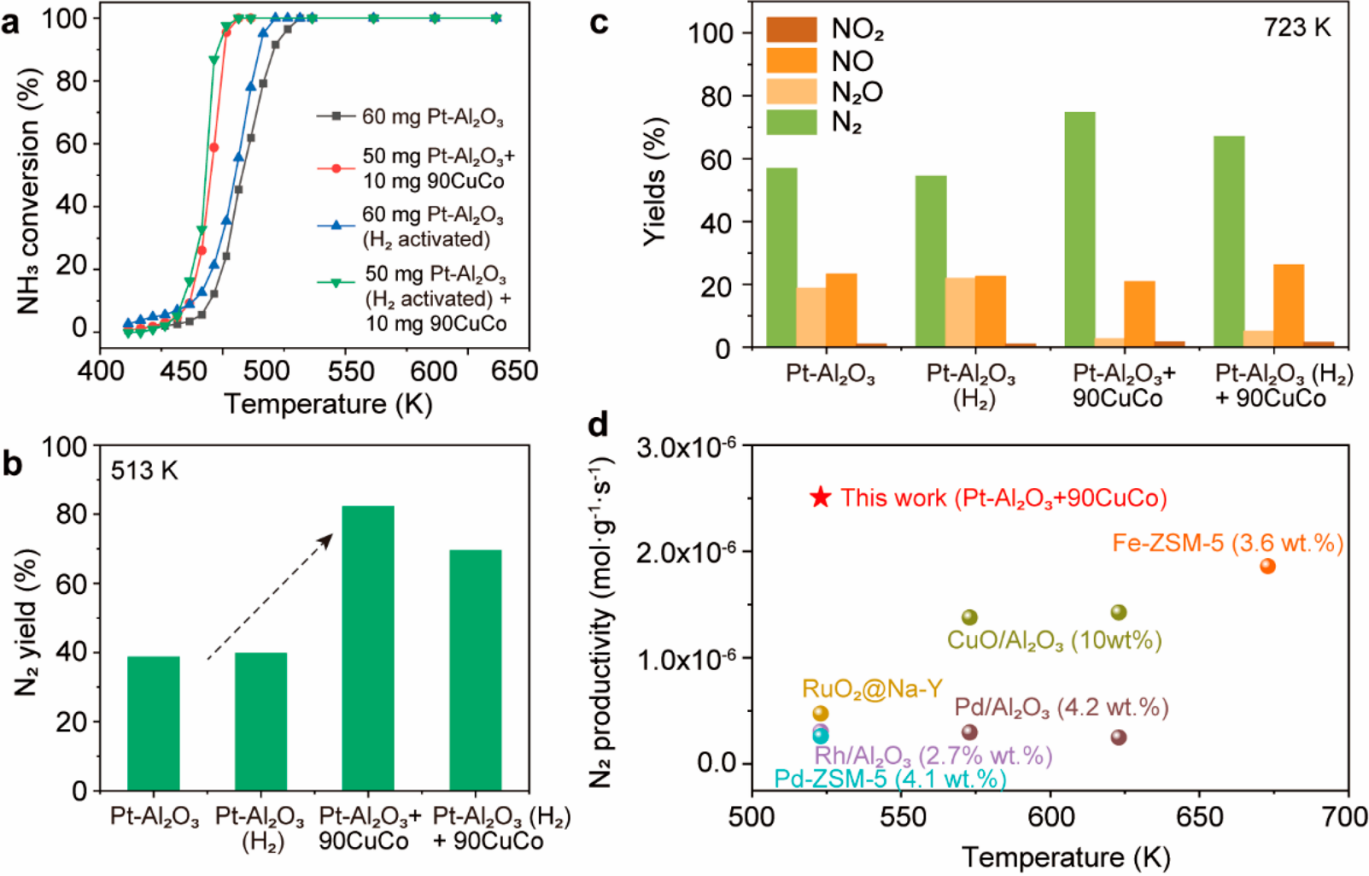
Catalysis performance of Pt–Al_2_O_3_ + 90CuCo catalysts. (a) NH_3_ conversion as the function of temperature. (b) N_2_ yield for 60 mg of 1 wt % Pt–Al_2_O_3_, 60 mg of 1 wt % Pt–Al_2_O_3_ (H_2_-activation), 50 mg of 1 wt % Pt–Al_2_O_3_ + 10 mg of 90CuCo, and 50 mg of 1 wt % Pt–Al_2_O_3_ (H_2_-activation) + 10 mg of 90CuCo catalysts at 513 K. (c) Yields of N_2_, N_2_O, NO, and NO_2_ for 60 mg of 1 wt % Pt–Al_2_O_3_, 50 mg of 1 wt % Pt–Al_2_O_3_ + 10 mg of 90CuCo, and 50 mg of 1 wt % Pt–Al_2_O_3_ (H_2_-activation) + 10 mg of 90CuCo catalysts at 723 K. (d) Comparison of N_2_ productivity for 50 mg of 1 wt % Pt–Al_2_O_3_ + 10 mg of 90CuCo with other catalysts. Reaction conditions are as follows: *T*_bed_ = 423–723 K, *m*_cat_ = 60 mg, 5000 ppm of NH_3_, 5% O_2_ balanced in He, gas flow of 100 mL·min^–1^, and WHSV = 500 mL_NH3_·h^–1^·g^–1^.

In light of the excellent N_2_O elimination ability of CuO–Co_3_O_4_ catalysts, we hypothesize that adding 90CuCo into the 1 wt % Pt–Al_2_O_3_ catalyst will inhibit the release of N_2_O and improve N_2_ selectivity at high temperatures. To prove it, the catalytic performance of 60 mg of Pt–Al_2_O_3_, 60 mg of Pt–Al_2_O_3_ (H_2_-activated), and 50 mg of Pt–Al_2_O_3_ + 10 mg of 90CuCo catalysts were compared (for 5000 ppm of NH_3_–SCO). The Pt–Al_2_O_3_ and 90CuCo were physically mixed. After the 50 mg of Pt/Al_2_O_3_ and 10 mg of 90CuCo were weighed, the two powders were ground in a mortar and pestle for a few minutes until 90CuCo was uniformly dispersed. The addition of just 10 mg of 90CuCo shifted the conversion to the low-temperature region by 50 K (Figure 7a). H_2_ activation slightly improves the activity of Pt–Al_2_O_3_, while the activity is still below that of Pt–Al_2_O_3_ + 90CuCo. With the addition of 90CuCo, complete NH_3_ conversion is achieved at 513 K, resulting in an increase in the N_2_ yield of approximately 40% (Figure 7b). At 723 K, 75% N_2_ selectivity is achieved after adding 90CuCo, which is much higher than that of pure Pt–Al_2_O_3_ (55%) and H_2_-activated Pt–Al_2_O_3_ (53%). The NO and NO_2_ yields are similar after adding 90CuCo, while the release of N_2_O is significantly suppressed (Figure 7c). N_2_O-TPD demonstrated that 90CuCo adsorbs N_2_O more readily than Pt/Al_2_O_3_ (Figure S32). Therefore, N_2_O released from the Pt/Al_2_O_3_ surface can be adsorbed and decomposed by 90CuCo. These results suggest that 90CuCo can enhance the low-temperature activity and help to remove N_2_O at high temperatures. Comparing the performance of the Pt–Al_2_O_3_ + 90CuCo with literatures,^69−72^ we achieved at least five times higher N_2_ productivity than other noble metal systems at 523 K (Figure 7d). Herein, the presented CuO–Co_3_O_4_ system provides a new strategy for increasing activity with the inhibited release of N_2_O in the selective NH_3_ oxidation.

## 4 Conclusions

Our work studied the rarely reported N_2_–N_2_O–N_2_ formation as a function of temperature in the selective ammonia oxidation reaction. In general, the selectivity of N_2_ decreases with increasing temperature, forming byproducts N_2_O or NO. To mitigate the NO_*x*_ formation, CuO–Co_3_O_4_ catalysts can convert the as-formed N_2_O to N_2_ due to the formation of the Co(II)–V_O_–Cu(I) intermediate species, as proven in the *in situ* XAFS/NEXAFS studies. The O_2_ activation ability of Co_3_O_4_ is the key to the formation of N_2_O, while the redox of CuO–Co_3_O_4_ interfaces promotes the dissociation of the N–O bond of N_2_O, forming N_2_ and active O species. Here, we proposed a de-N_2_O mechanism for N_2_ formation over such a bifunctional surface. The rate of N_2_O generation and decomposition can be controlled by adjusting the ratio of surface CuO and Co_3_O_4_, thereby changing the product selectivity. 90 wt % CuO–Co_3_O_4_ has similar N_2_O generation and conversion rates. A 120 K operation window (N_2_ yield >90%) from 553 to 673 K is achieved, which is wider than most of the literature. This ability to remove N_2_O at high temperatures can be further exploited to reduce the N_2_O emissions from noble metal catalysts. Adding 90 wt % CuO–Co_3_O_4_ catalyst to Pt–Al_2_O_3_ can effectively improve the low-temperature activity and reduce the selectivity of N_2_O by 17% at high temperatures. This work develops a solution to address N_2_O release and provides a fundamental understanding toward the formation and removal of N_2_O for selective oxidation reactions in the chemical industry.

## Supporting Information Available

NAP-NEXAFS spectra, DFT calculation results, HAADF-STEM images, XRD, Cu K edge and Co K edge XANES spectra. *k*^3^-weighted EXAFS oscillations, EXAFS fit parameters, reaction profiles, NH_3_–SCO performances of catalysts in the literature, and *in situ* XAFS spectra (PDF)
