# Supplementary material for: Cascade NH3 Oxidation and N2O Decomposition via Bifunctional Co and Cu Catalysts
Source: ACS Catal. 2023 Oct 12;13(20):13816–27. doi: 10.1021/acscatal.3c02392 (PMC10594585; doi:10.1021/acscatal.3c02392)
Supplement: Supplementary file 1 — cs3c02392_si_001.pdf [file cs3c02392_si_001.pdf]

Supporting Information

Cascade NH_3 oxidation and N_2O reduction *via* bifunctional Co and Cu catalysts

Xuze Guan,[1] Hiroyuki Asakura,[2,3] Rong Han,[4] Siyuan Xu,[4] Hao-Xin Liu,[5] Lu Chen,[1] Zhangyi Yao,[1] Jay Hon Cheung Yan,[1]* Tsunehiro Tanaka,[3] *Yuzheng Guo,[4] Chun-Jiang Jia,[5] Feng Ryan Wang[1*]*

1. Department of Chemical Engineering, University College London, Roberts Building, Torrington Place, London *WC1E 7JE*, UK
2. Department of Applied Chemistry, Faculty of Science and Engineering, Kindai University 3-4-1, Kowakae, Higashi-Osaka, Osaka, 577-8502, Japan
3. Department of Molecular Engineering, Graduate School of Engineering, Kyoto University, Kyotodaigaku Katsura, Nishikyo-ku, Kyoto 615-8510, Japan
4. School of Electrical Engineering and Automation, Wuhan University, Wuhan, 430072, China
5. Key Laboratory for Colloid and Interface Chemistry, Key Laboratory of Special Aggregated Materials, School of Chemistry and Chemical Engineering, Shandong University, Jinan, 250100, China

*e-mail: ryan.wang@ucl.ac.uk; asakura@apch.kindai.ac.jp

Text S1

The decomposition of N_2O is reflected in the fact that in the presence of a significant amount of surface N_2O at both 573 K and 673 K, there is significantly less N_2O release and more N_2 generation at 673 K (Figure 4). This interpretation was based on the understanding that N_2O decomposition becomes more favourable at higher temperatures. As the reaction temperature increases, the energy barrier for N_2O decomposition is surmounted more easily, resulting in an elevated rate of N_2O breakdown into N_2 and reactive O. Therefore, the fact that N_2O signals are still detectable at 400 °C implies that N_2O is indeed being formed but subsequently decomposed as well, indicating a more intricate dynamic. This makes it not possible to quantify the amount of N_2O that is produced and the amount of N_2O that is decomposed.

The reactions for the formation of N_2 are shown below:

$$2NH_3 + 2O_2 \rightarrow N_2O + 3H_2O \qquad \text{Equation 1}$$

$$N_2O \rightarrow N_2 + 1/2O_2 \qquad \text{Equation 2}$$

$$2NH_3 + 3/2O_2 \rightarrow N_2 + 3H_2O \qquad \text{Equation 3}$$

The de-N_2O mechanism includes two steps: First, ammonia is oxidised to N_2O (Eq. 1). Second, the N_2 is formed when the as-prepared N_2O decomposes (Eq. 2). Also, NH_3 can be oxidised to N_2 bypassing N_2O (Eq. 3). The reaction rate in Eq.1-3 is defined as $r1$, $r2$ and $r3$, respectively. Based on this two-step mechanism, high selectivity towards N_2 can only be achieved at $r2 = r1$ conditions. At low temperatures, significant N_2O emissions can be observed, indicating that $r1 > r2$. Increasing temperatures, the N_2 selectivity increases. There are two possible reasons: 1) $r1$ decreases, leading to less production of N_2O and $r3$ increases; 2) $r2$ increases to a similar level of $r1$. However, to our best knowledge, increasing temperature does not favour increased N_2 selectivity ($r3$) by other mechanisms.

Shishido et al.[1] reported that Ag nanoparticles (Ag NPs) over Al_2O_3 form much more N_2O by-products than highly dispersed Ag species (Ag HDs) over Al_2O_3. The metallic-state Ag on the Ag NPs/Al_2O_3 catalyst has a stronger O_2 activation ability than the cationic Ag on Ag HDs/Al_2O_3, therefore, a more active [O] is formed and consumed on Ag NPs/Al_2O_3. This accelerates the formation N_2O by-product. According to the results of NH_3-DRIFTS, they claimed that the surface amounts of NO_{3ads} and NH_{2ads} intermediates over Ag HDs/Al_2O_3 were much higher than those over Ag NPs/Al_2O_3, which further facilitated the formation of the N═N–M intermediate and N═N–M itself further decomposed to N_2. N═N–M could decompose to N_2 at low temperatures, or it could further transform to the N═N–O–M intermediate at high temperatures. The N═N–O–M intermediate further decomposed to N_2O over the catalyst's surface. However, the N_2 selectivity for the Ag NPs/Al_2O_3 catalyst also increases at high temperatures, which was not discussed and cannot explained by the increased formation of N═N–M.

For catalysts following the i-SCR mechanism, the formation of nitrate species can be observed[2]. He and colleagues[3] reported that as the temperature further rose from 250 to 350 °C, NH_3 adsorption rapidly decreased, accompanied by the gradual formation of nitrates

(1545 and 1250 cm^{-1}) on the surface of 10 wt% Ag/Al_2O_3 catalyst. At the same time, N_2 production continuously increased and remained at a high level, whereas N_2O formation gradually decreased. In the high-temperature region (above 350 °C), only nitrate species were observed on the catalyst surface.

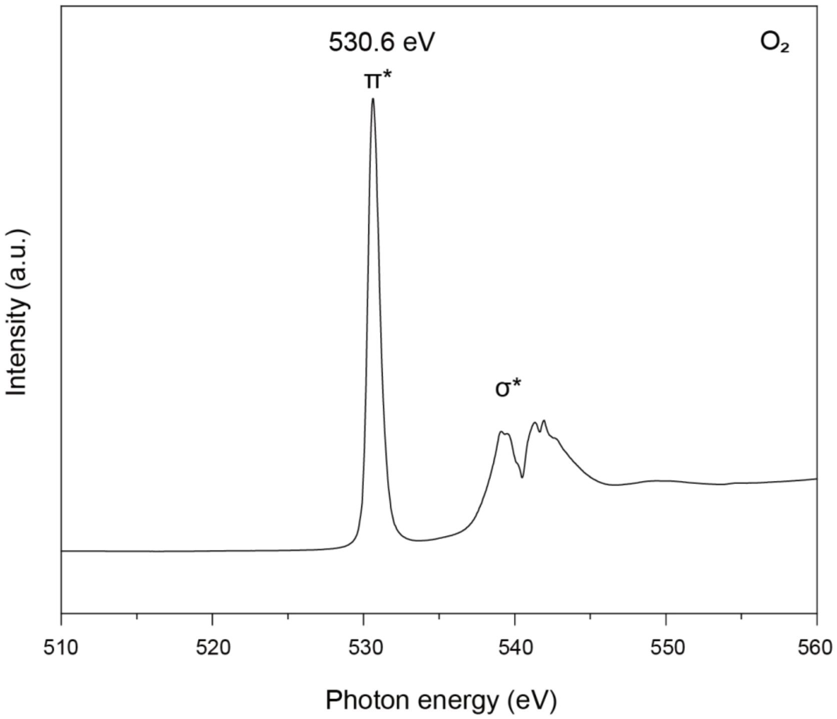

Figure S1. O K edge near edge X-ray absorption fine structure (NEXAFS) spectrum of gaseous O_2.

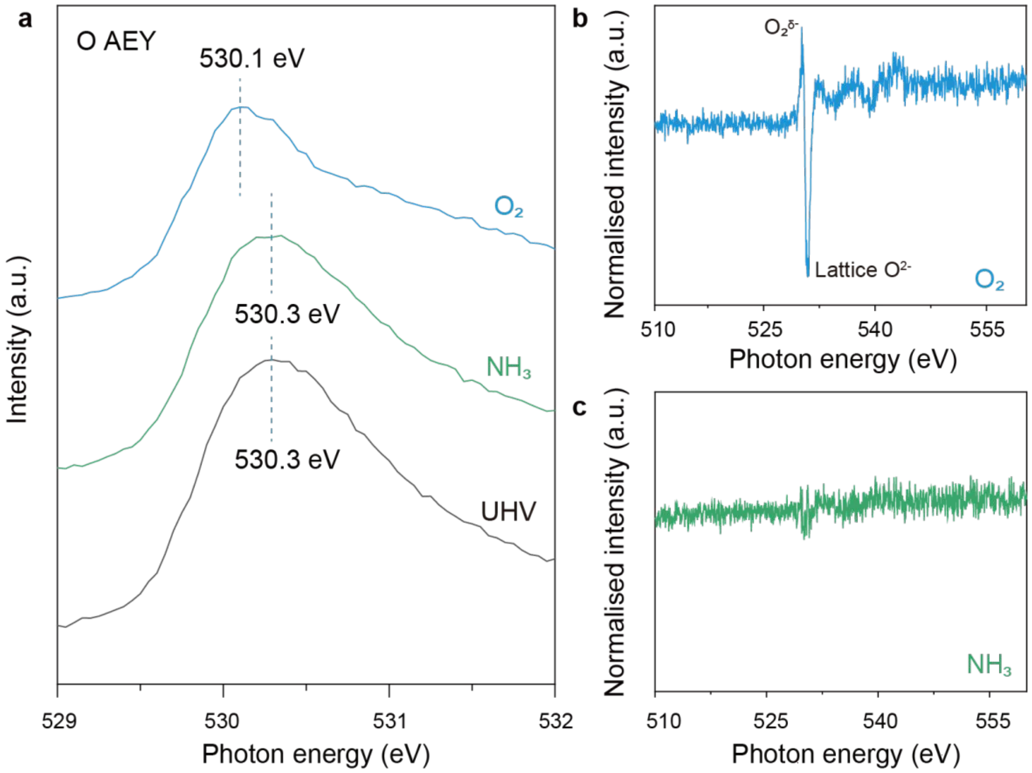

Figure S2. a O K edge NEXAFS spectra (Auger electron yield (AEY) mode) of Co_3O_4 under UHV, NH_3 and O_2 at room temperature. **b** Spectral differences between UHV and O_2 conditions at room temperature. **c** Spectral differences between UHV and NH_3 conditions at room temperature.

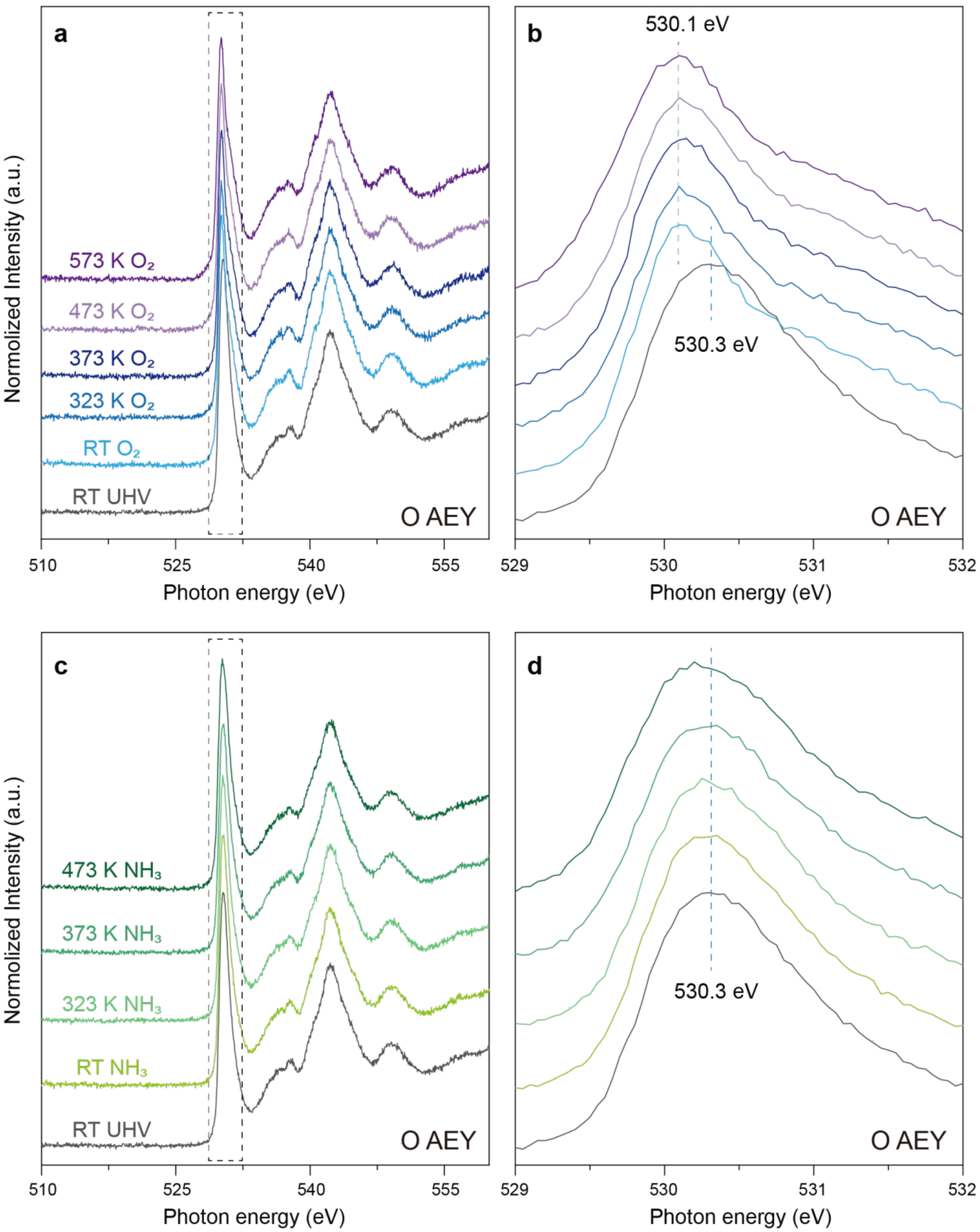

Figure S3. O K edge NEXAFS spectra (AEY mode) of Co_3O_4 under **a,b** O_2 and **c,d** NH_3 at various temperature.

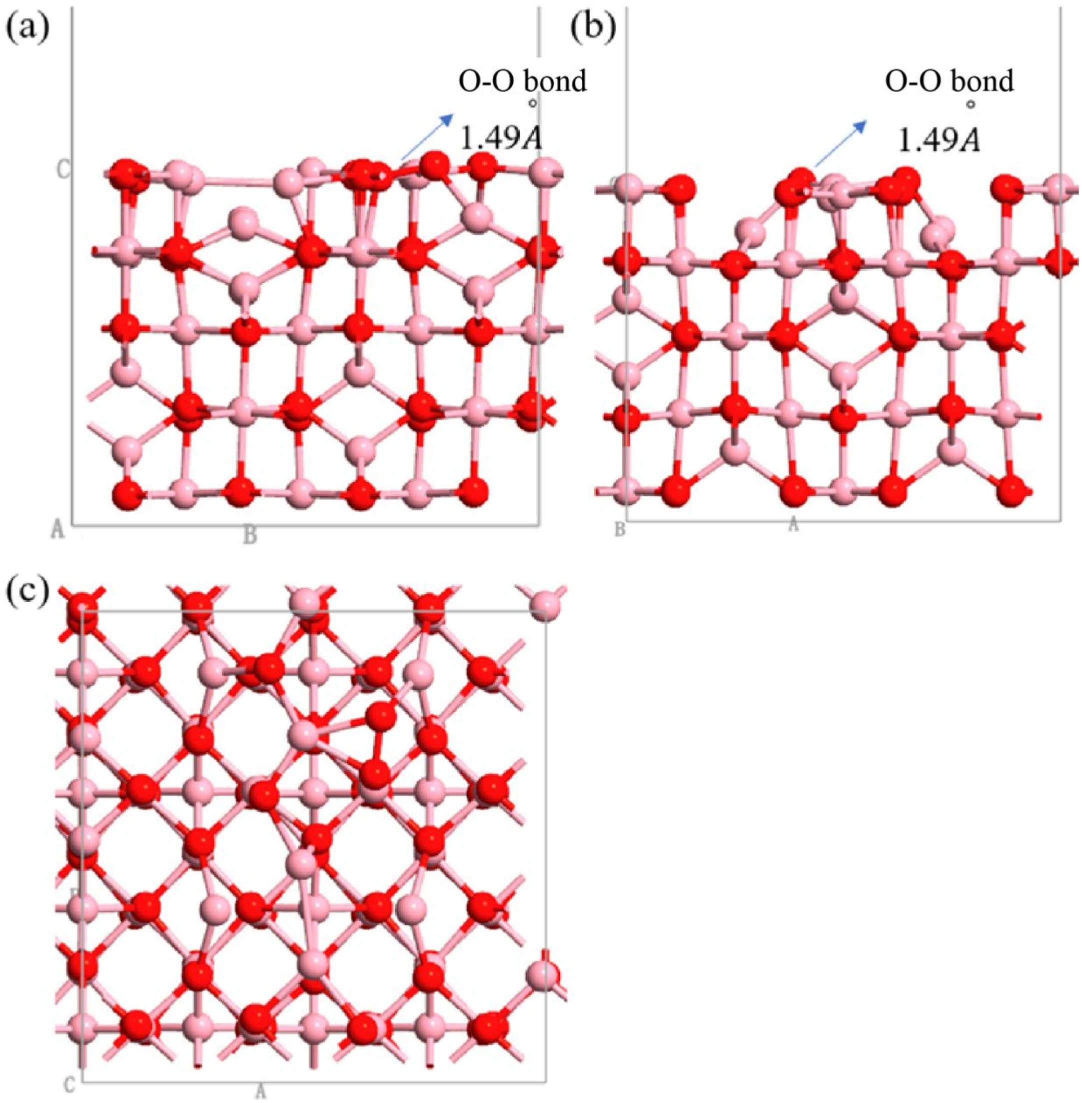

Figure S4. DFT calculation of O_2 adsorption on Co_3O_4 (100) surface. The (**a**) front, (**b**) side and (**c**) overview of the O_2 molecule adsorption on Co_3O_4 (100) surface. Co atoms are pink and O atoms are red.

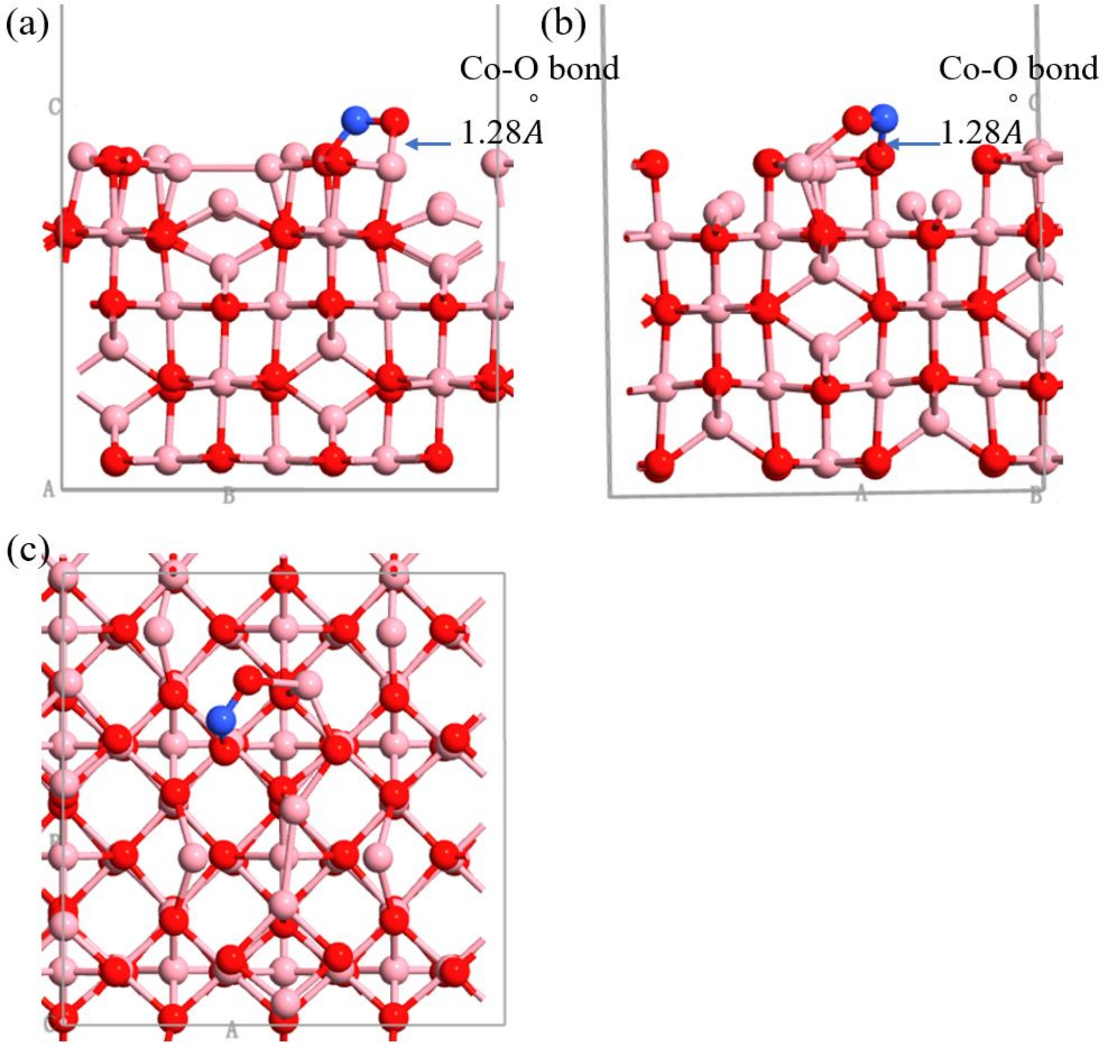

Figure S5. DFT calculation of NO adsorption on Co_3O_4 (100) surface. The (**a**) front, (**b**) side and (**c**) overview of the NO molecule adsorption on Co_3O_4 (100) surface. Co atoms are pink, O atoms are red and N atoms are blue.

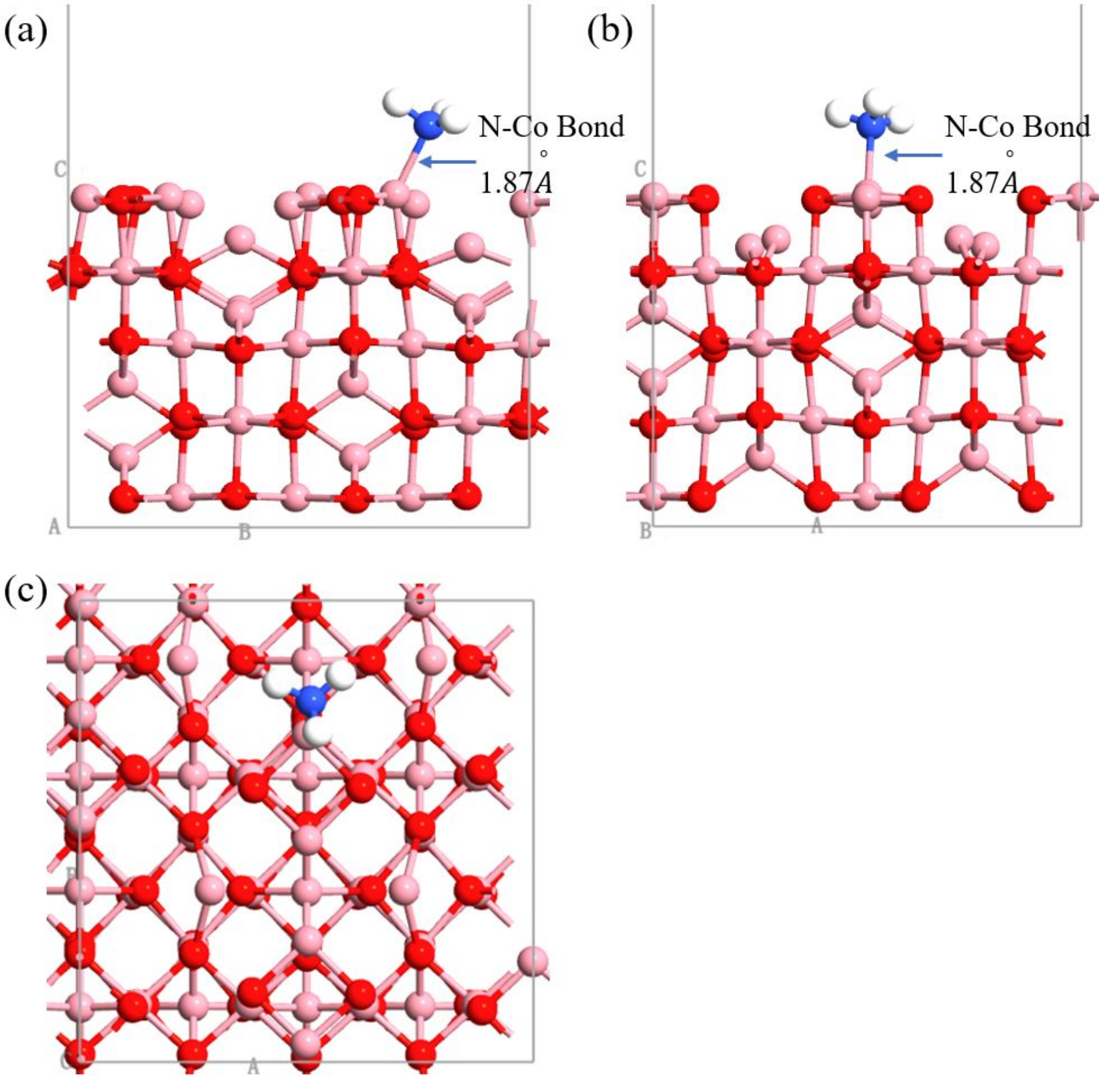

Figure S6. DFT calculation of NH_3 adsorption on Co_3O_4 (100) surface. The (**a**) front, (**b**) side and (**c**) overview of the NH_3 molecule adsorption on Co_3O_4 (100) surface. Co atoms are pink, O atoms are red, H atoms are white and N atoms are blue.

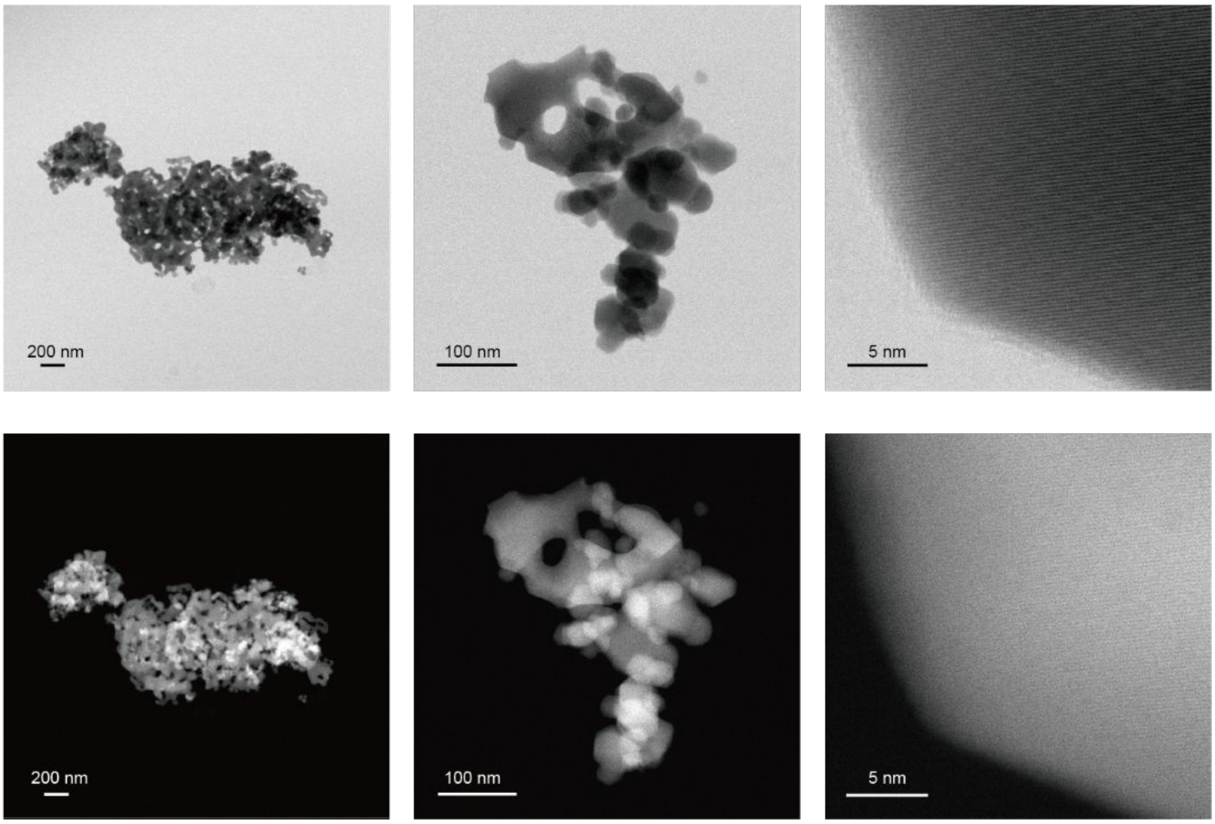

Figure S7. Scanning transmission electron microscopy (STEM) images of pure Co_3O_4.

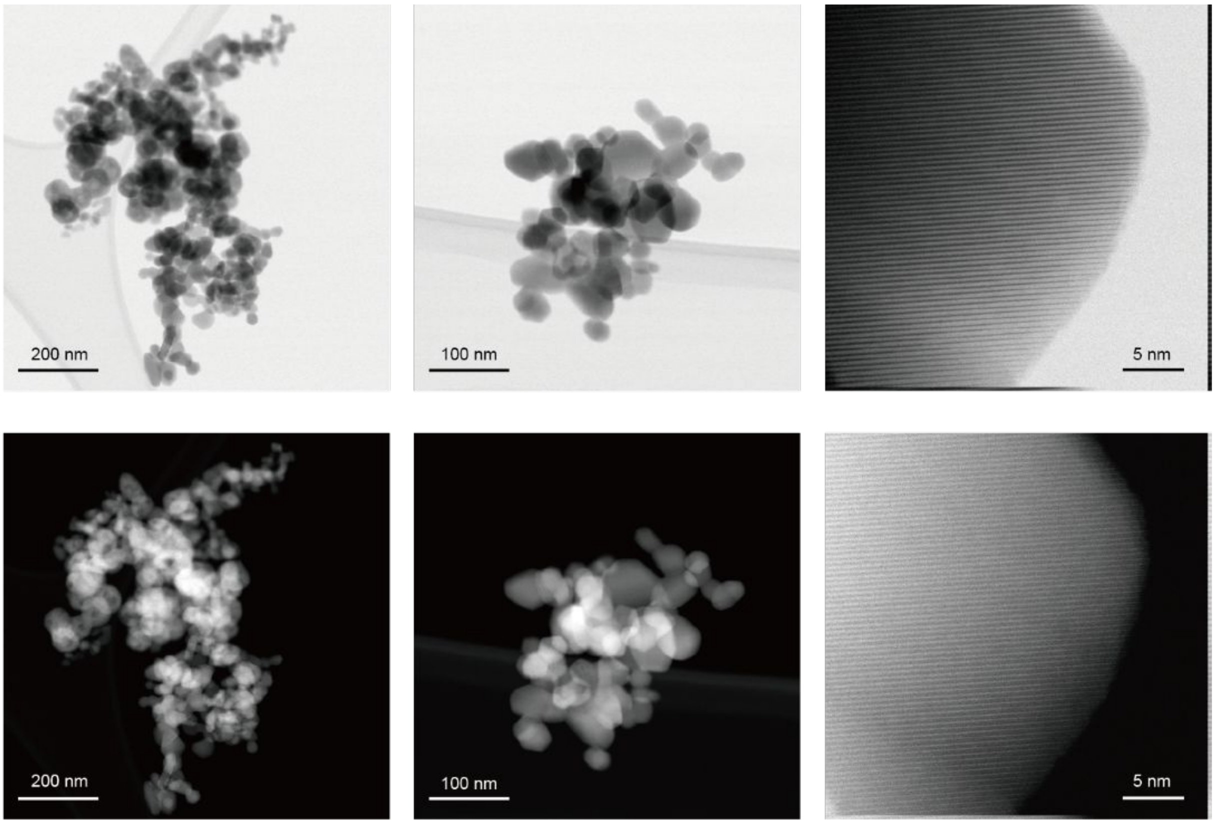

Figure S8. STEM images of 1wt% CuO-Co_3O_4.

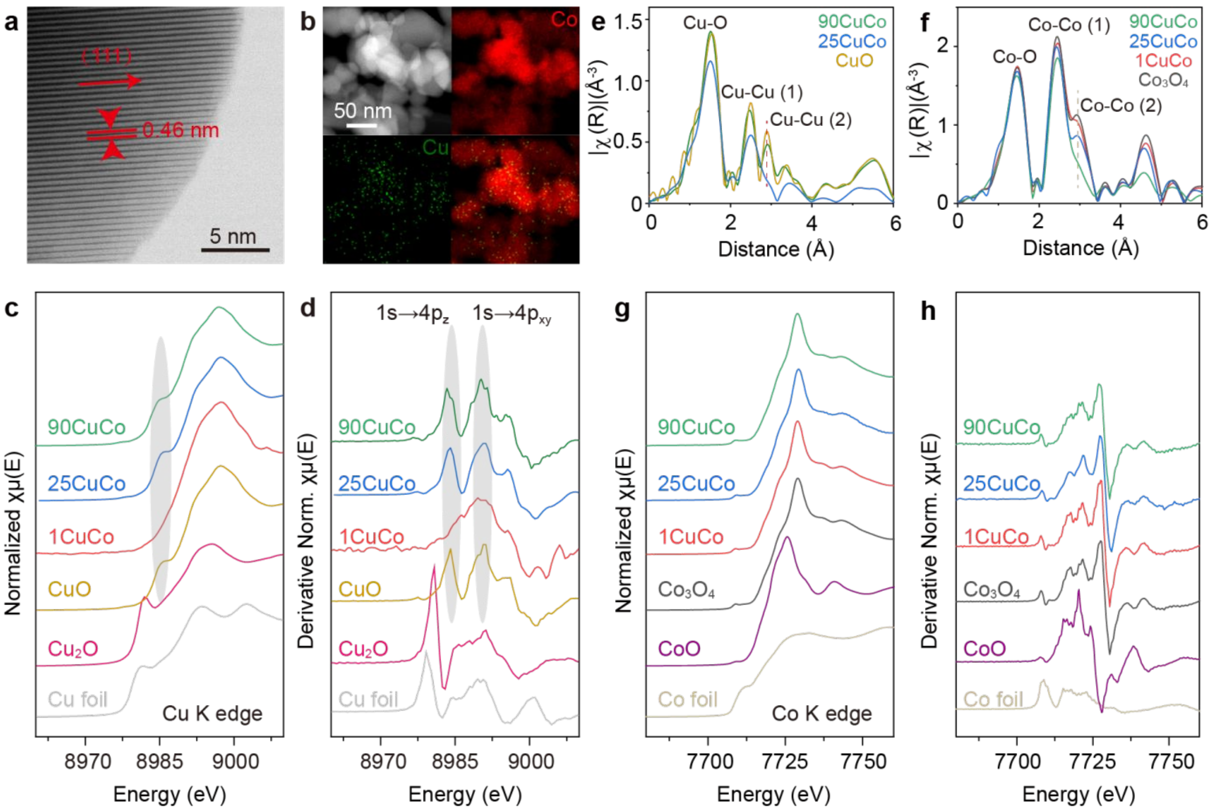

Figure S9. Characterization of CuO-Co_3O_4 catalysts. a, Bright field (BF)-STEM image for 1wt% CuO on Co_3O_4. **b,** Energy dispersive X-ray (EDX) mapping of 1wt% CuO on Co_3O_4. **c,** Cu K-edge XANES spectra of CuO-Co_3O_4 with various CuO loading. **d,** First derivative of Cu K-edge XANES spectra of CuO-Co_3O_4 with various CuO loading. **e,** Cu K-edge EXAFS spectra of CuO-Co_3O_4 with various CuO loading. **f,** Co K-edge EXAFS spectra of CuO-Co_3O_4 with various CuO loading. **g,** Co K-edge XANES spectra of CuO-Co_3O_4 with various CuO loading. **h,** First derivative of Co K-edge XANES spectra of CuO-Co_3O_4 with various CuO loading.

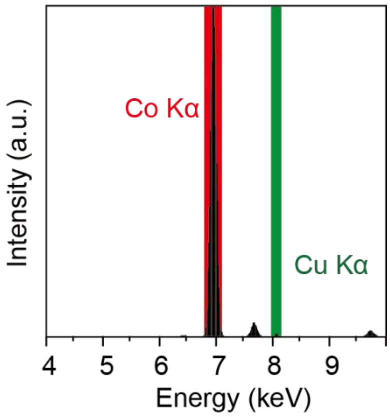

Figure S10. EDX spectra of CuO-Co_3O_4 with 1wt% CuO loading. Collected from same areas in Figure 9b. Peaks in the red region and green region correspond to Co Kα, Cu Kα, respectively.

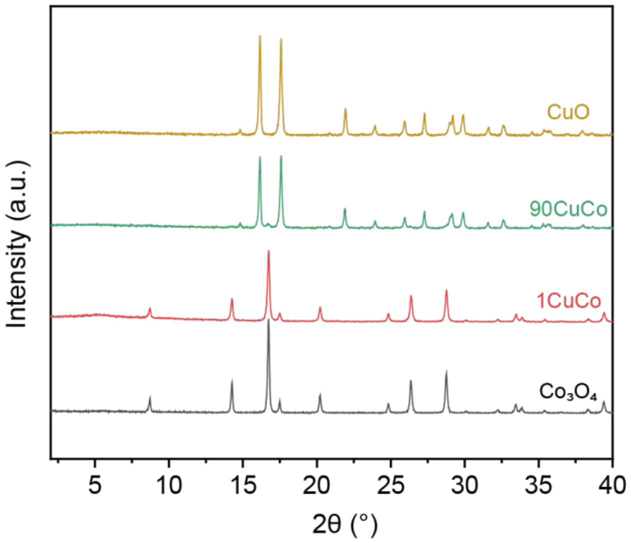

Figure S11. X-ray diffraction (XRD) patterns of CuO-Co_3O_4 with various Cu loading. XRD of CuO, Co_3O_4 and CuO-Co_3O_4 with 1wt% and 90wt% CuO loading.

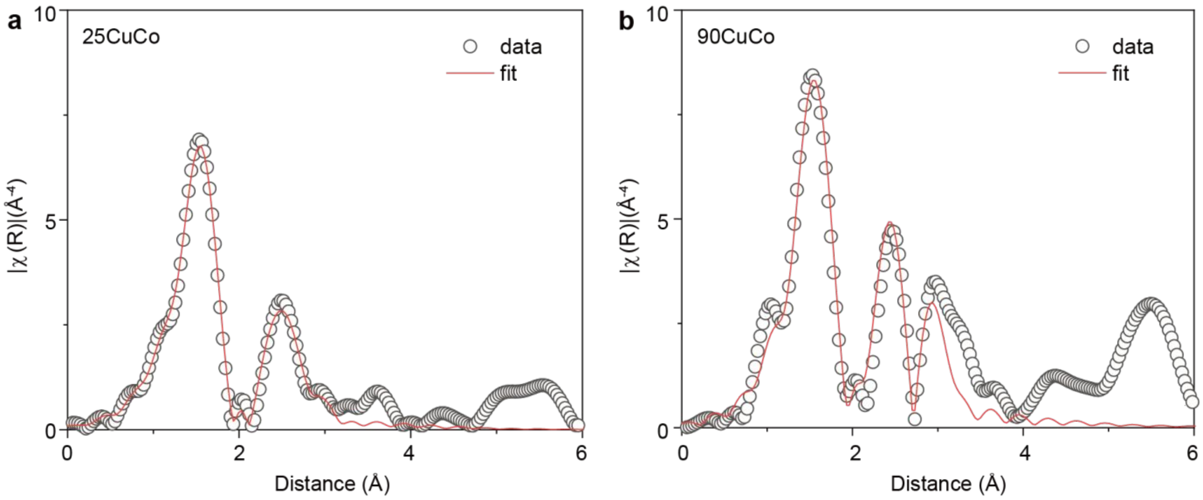

Figure S12. EXAFS fitting results of Cu K edge of CuO-Co_3O_4 catalysts with (**a**) 25wt% and (**b**) 90wt% CuO loading. The k^2-weighted Fourier Transform EXAFS data are shown together with fitting results (red curves).

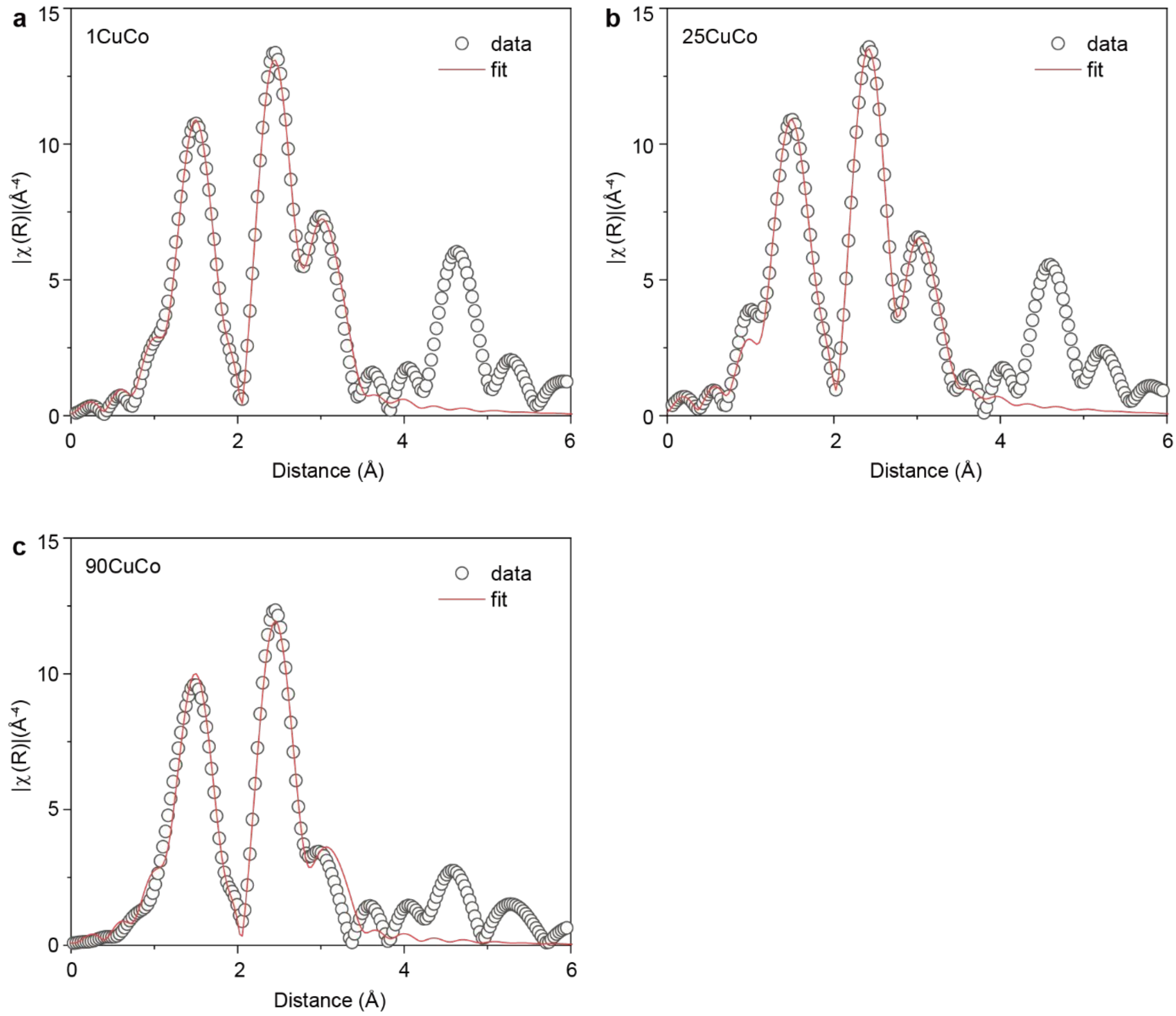

Figure S13. EXAFS fitting results of Co K edge of CuO-Co_3O_4 catalysts with (**a**) 1wt%, (**b**) 25wt% and (**c**) 90wt% CuO loading. The k^2-weighted Fourier Transform EXAFS data are shown together with fitting results (red curves).

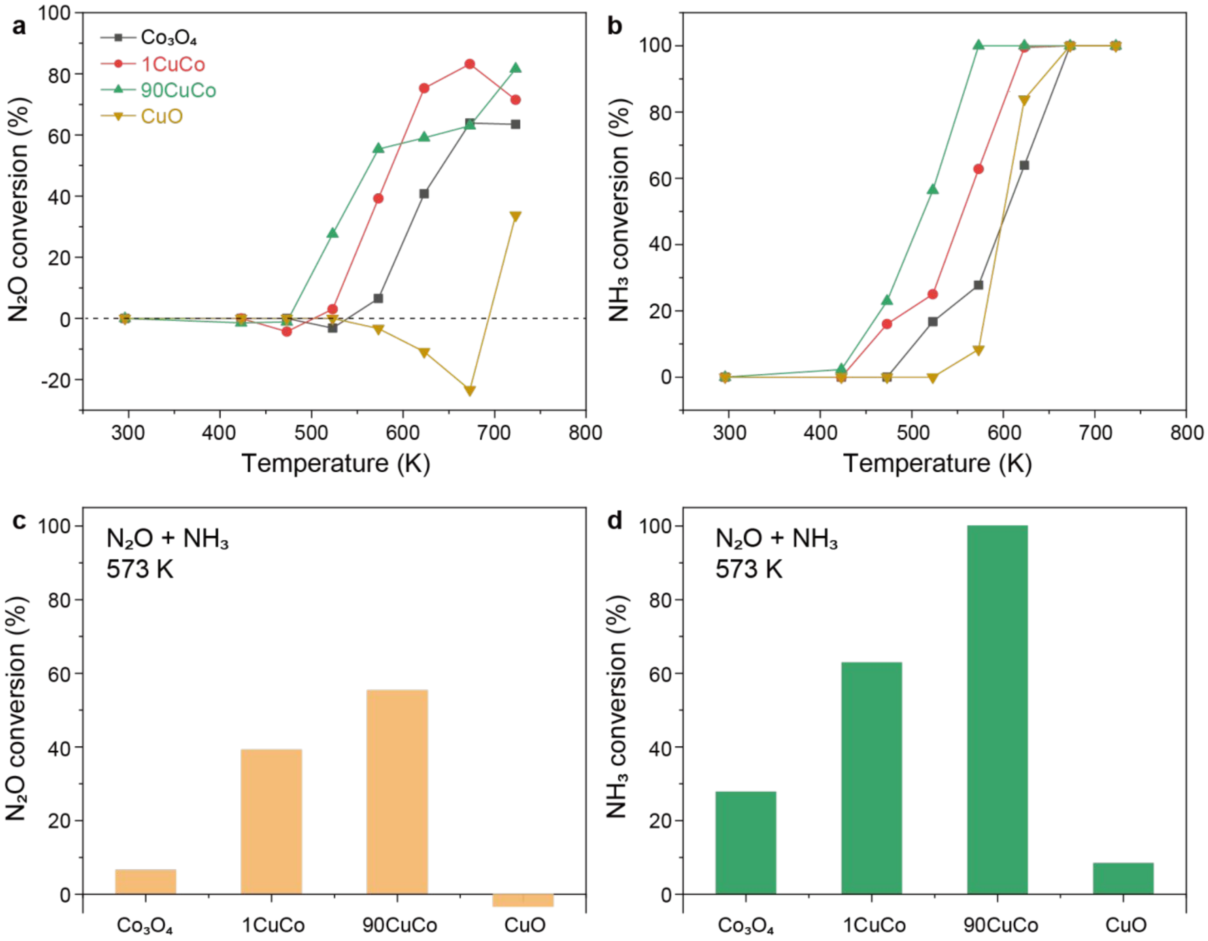

Figure S14. Catalytic performance in N_2O reduction by NH_3. Conversion profile for (**a**) N_2O and (**b**) NH_3 as function of temperature. **c,** The conversion of N_2O for CuO-Co_3O_4 catalysts at 573 K. **d,** The conversion of NH_3 for CuO-Co_3O_4 catalysts at 573 K. Reaction condition: 120 mg catalyst, 5000 ppm NH_3, 5000 ppm N_2O balanced in He, gas flow: 80 mL/min.

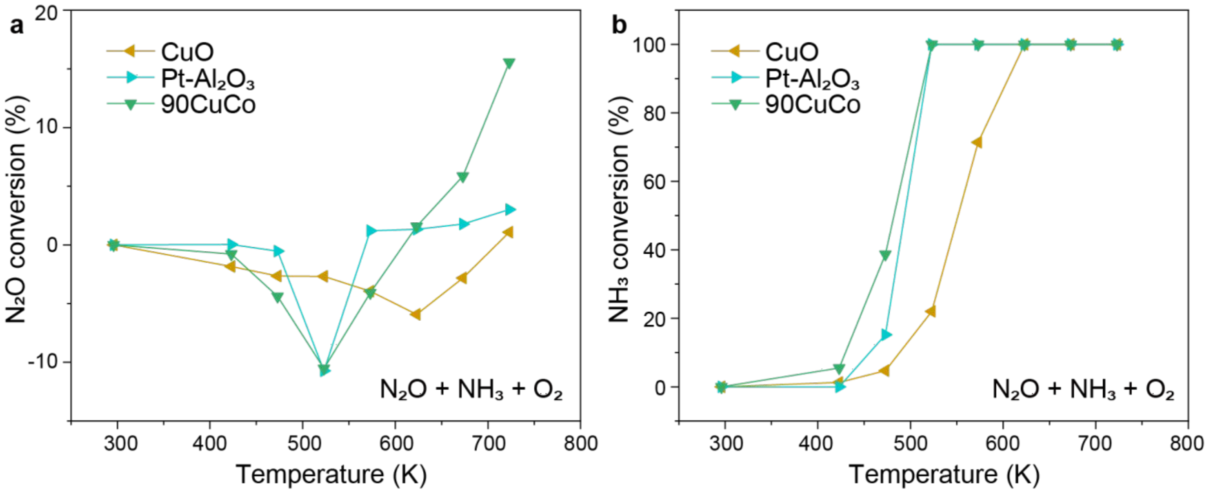

Figure S15. Catalytic performance in N_2O reduction by NH_3 in the presence of O_2. Conversion profile for (**a**) N_2O and (**b**) NH_3 as function of temperature. Reaction condition: 120 mg catalyst, 3000 ppm NH_3, 4500 ppm N_2O and 5% O_2 balanced in He, gas flow: 100 mL/min.

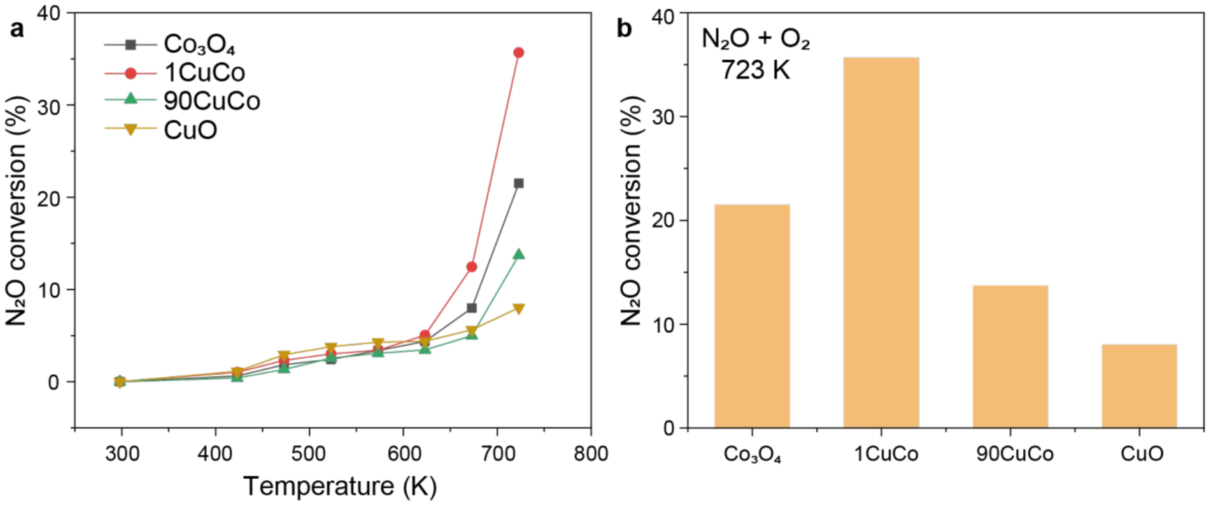

Figure S16. Catalytic performance in N_2O decomposition. **a,** Conversion profile for N_2O as function of temperature. **b,** The conversion of N_2O for pure Co_3O_4, 1wt% CuO-Co_3O_4, 90wt% CuO-Co_3O_4 and pure CuO at 723K. Reaction condition: 50 mg catalyst, 2500 ppm N_2O and 5% O_2 balanced in He, gas flow: 100 mL/min.

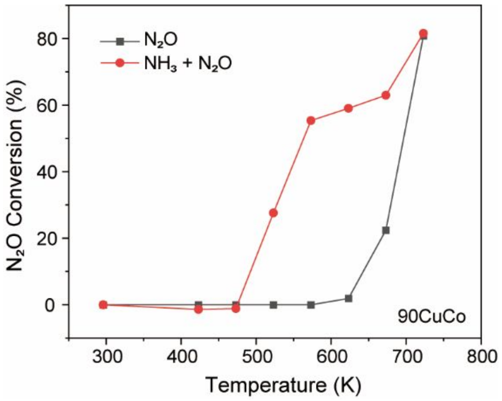

Figure S17. Catalytic performance in N_2O decomposition for 90CuCo with presence and absence of NH_3. Reaction condition: presence of NH_3 (120 mg catalyst, 5000 ppm NH_3, 5000 ppm N_2O balanced in He, gas flow: 80 mL/min) and absence of NH_3 (120 mg catalyst, 5000 ppm N_2O balanced in He, gas flow: 80 mL/min).

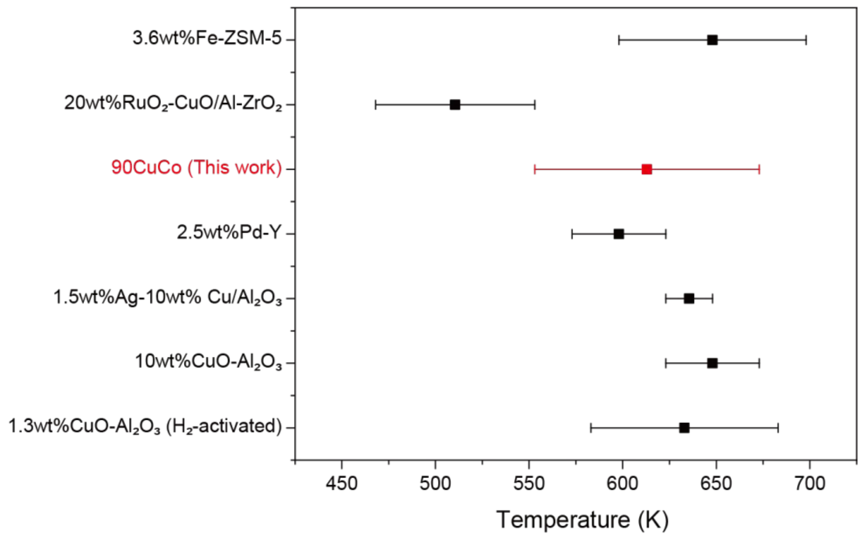

Figure S18. Comparison of operation window with the literature[4-9]. Operation window with 90% N_2 yield.

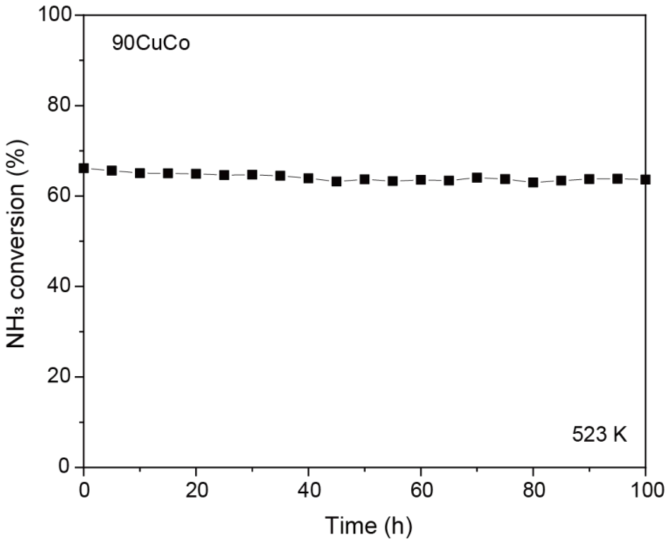

Figure S19. Long-term stability (100 h) test of 90CuCo catalyst for NH_3-SCO. Reaction conditions: m_{cat}=50 mg, 5000ppm NH_3, 5% O_2 balanced in He, gas flow: 100 mL/min, WHSV=600 $ml_{NH3}{\cdot}h^{-1}{\cdot}g^{-1}$, temperature: 523 K.

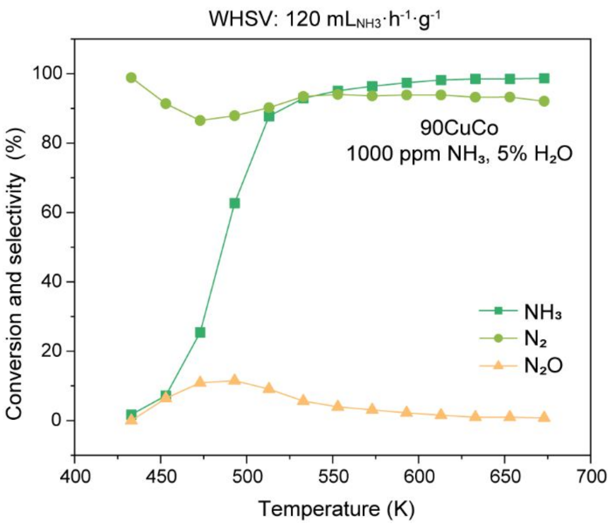

Figure S20. NH_3 conversion and product selectivity as the function of temperature for 90wt% CuO-Co_3O_4 catalyst in percentage (5%) concentrations of water.

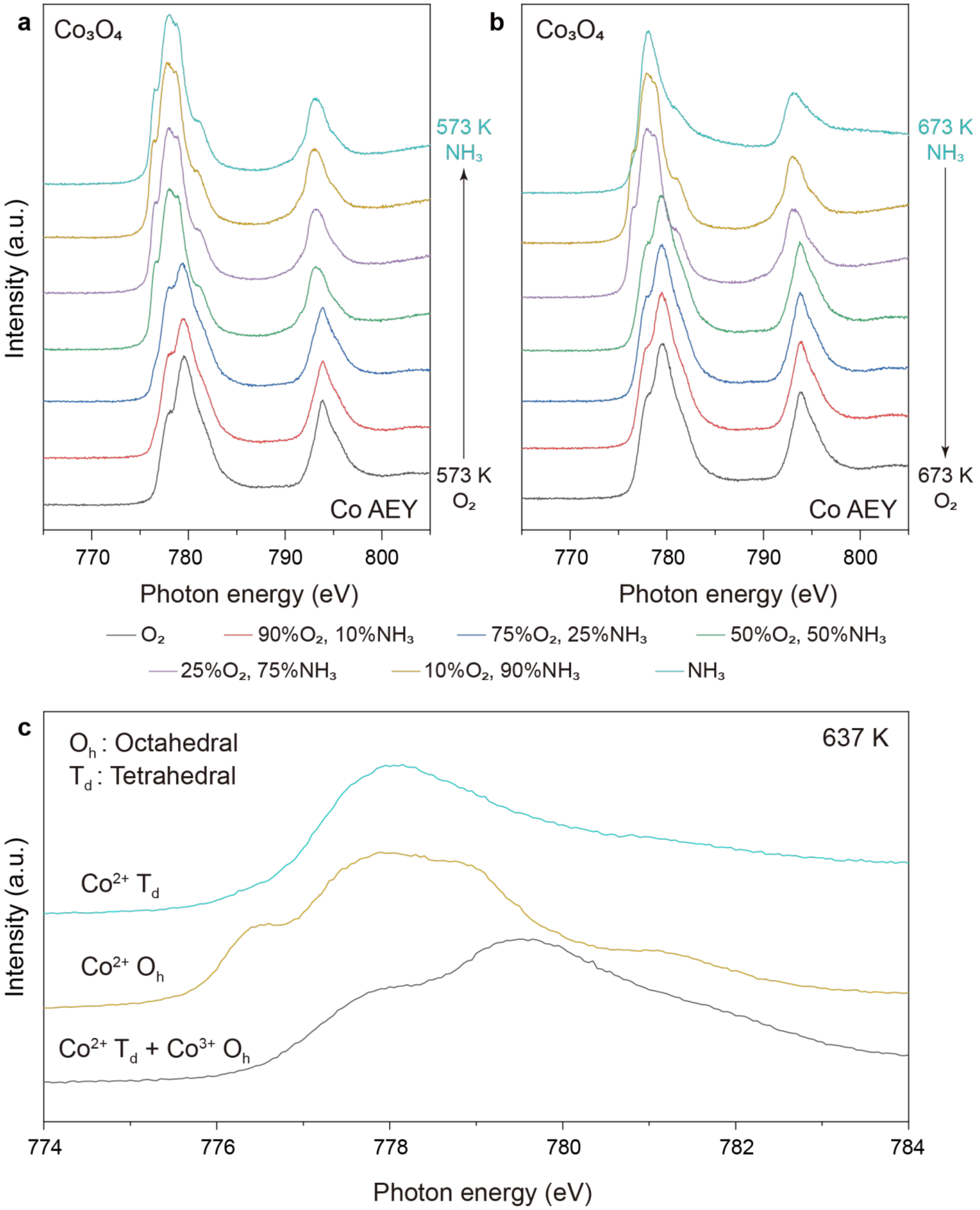

Figure S21. Near ambient pressure (NAP)-NEXAFS of Co_3O_4 under various gas conditions at 573K and 673 K. **a,** Co L-edge (AEY mode) of Co_3O_4 under various gas conditions at 573 K. **b,** Co L-edge (AEY mode) of Co_3O_4 under various gas conditions at 673 K. **c,** Comparison of the Co L-edge XAS and Co coordination of Co_3O_4 under different gas conditions at 673 K.

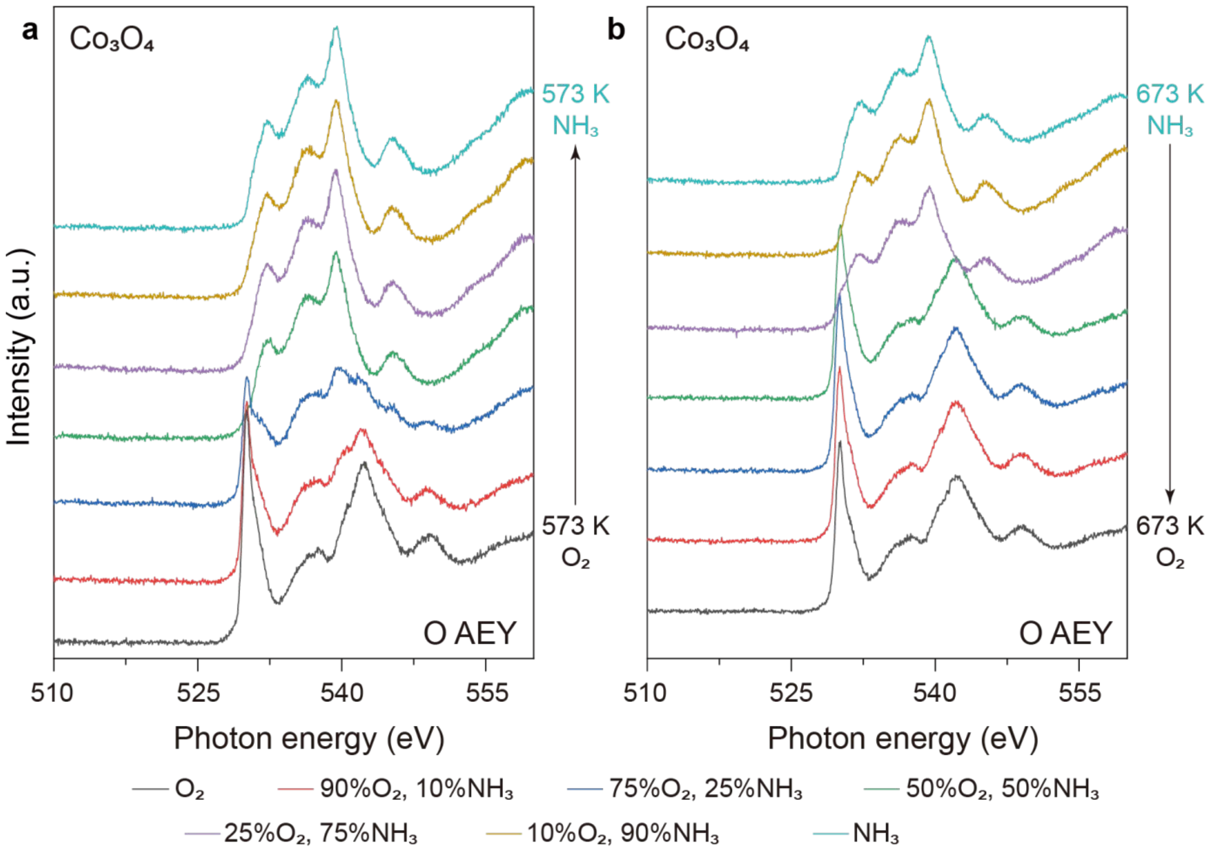

Figure S22. NAP-NEXAFS of Co_3O_4 under various gas conditions at 573K and 673 K. **a,** O K-edge (AEY mode) of Co_3O_4 under various gas conditions at 573 K. **b,** O K-edge (AEY mode) of Co_3O_4 under various gas conditions at 673 K.

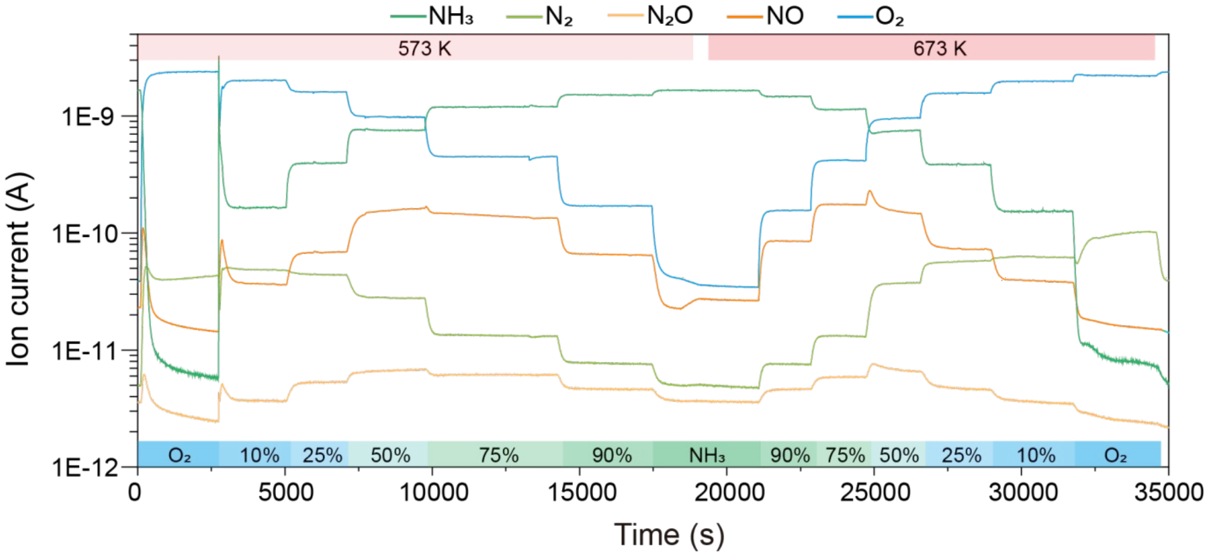

Figure S23. Mass spectrometry of NAP-NEXAFS experiments under various gas conditions at 573 K and 673 K

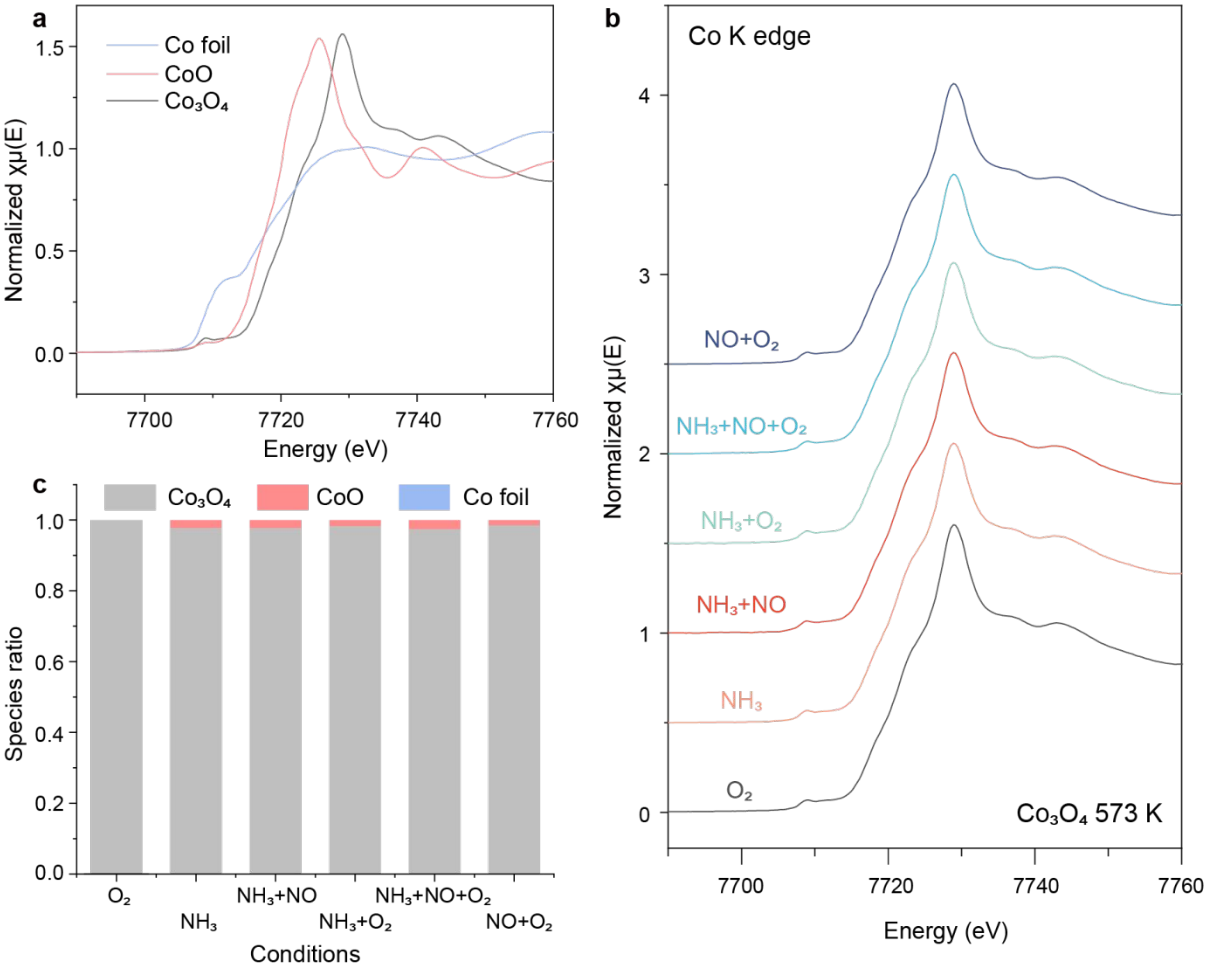

Figure S24. a, Co K edge X-ray Absorption Near Edge Structure (XANES) of Co foil (blue), CoO standard (red) and Co_3O_4 standard (black). **b,** *In situ* XANES spectra of Co_3O_4 under various gas conditions at 573 K. **c,** Co speciation of Co_3O_4 catalyst under O_2, NH_3, NH_3+NO, NH_3+NO+O_2, NH_3+O_2, NO+O_2 conditions at 573 K.

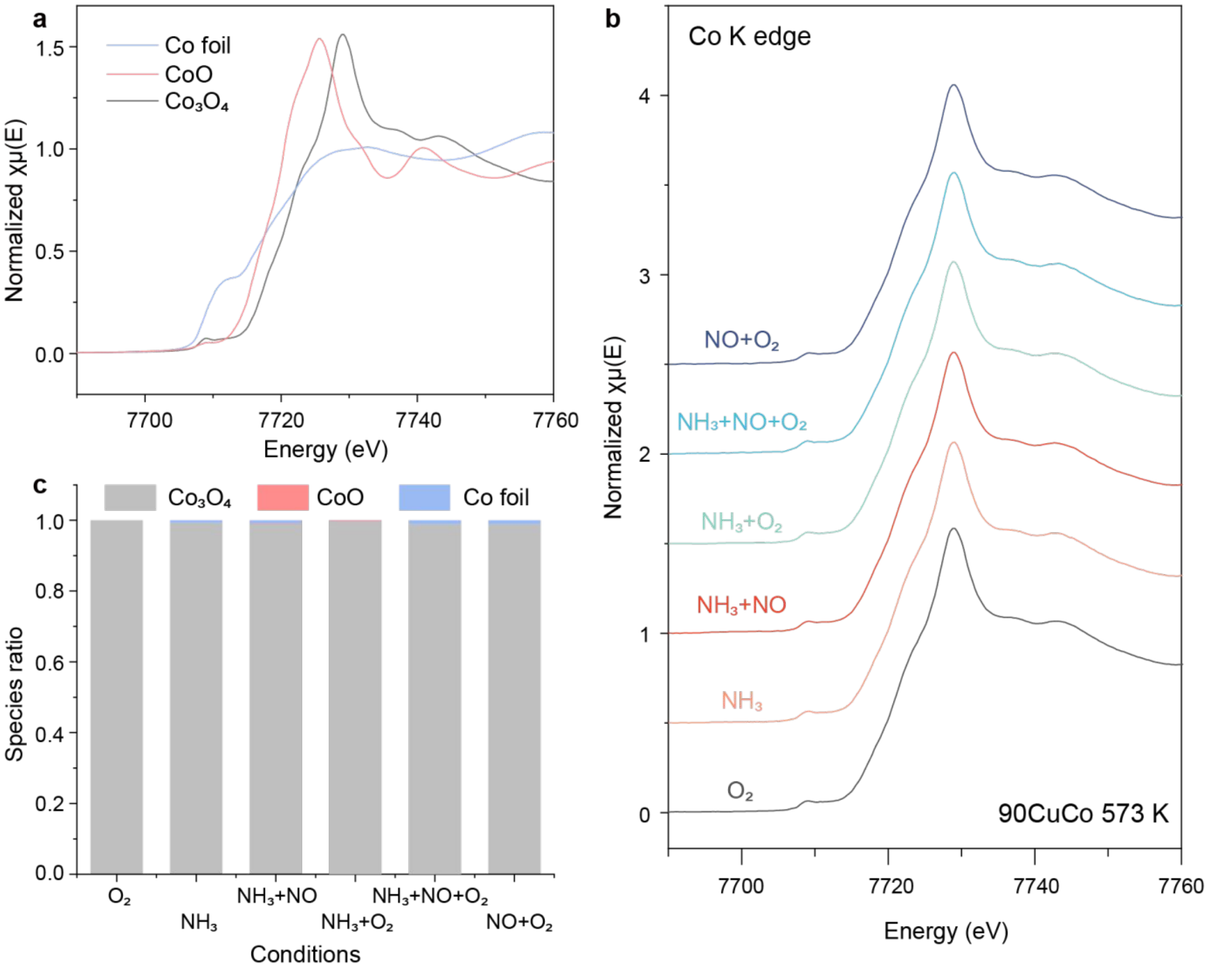

Figure S25. a, Co K edge XANES spectra of Co foil (blue), CoO standard (red) and Co_3O_4 standard (black). **b,** *In situ* XANES spectra of 90wt% CuO-Co_3O_4 under various gas conditions at 573 K. **c,** Co speciation of 90wt% CuO-Co_3O_4 catalyst under O_2, NH_3, NH_3+NO, NH_3+NO+O_2, NH_3+O_2, NO+O_2 conditions at 573 K.

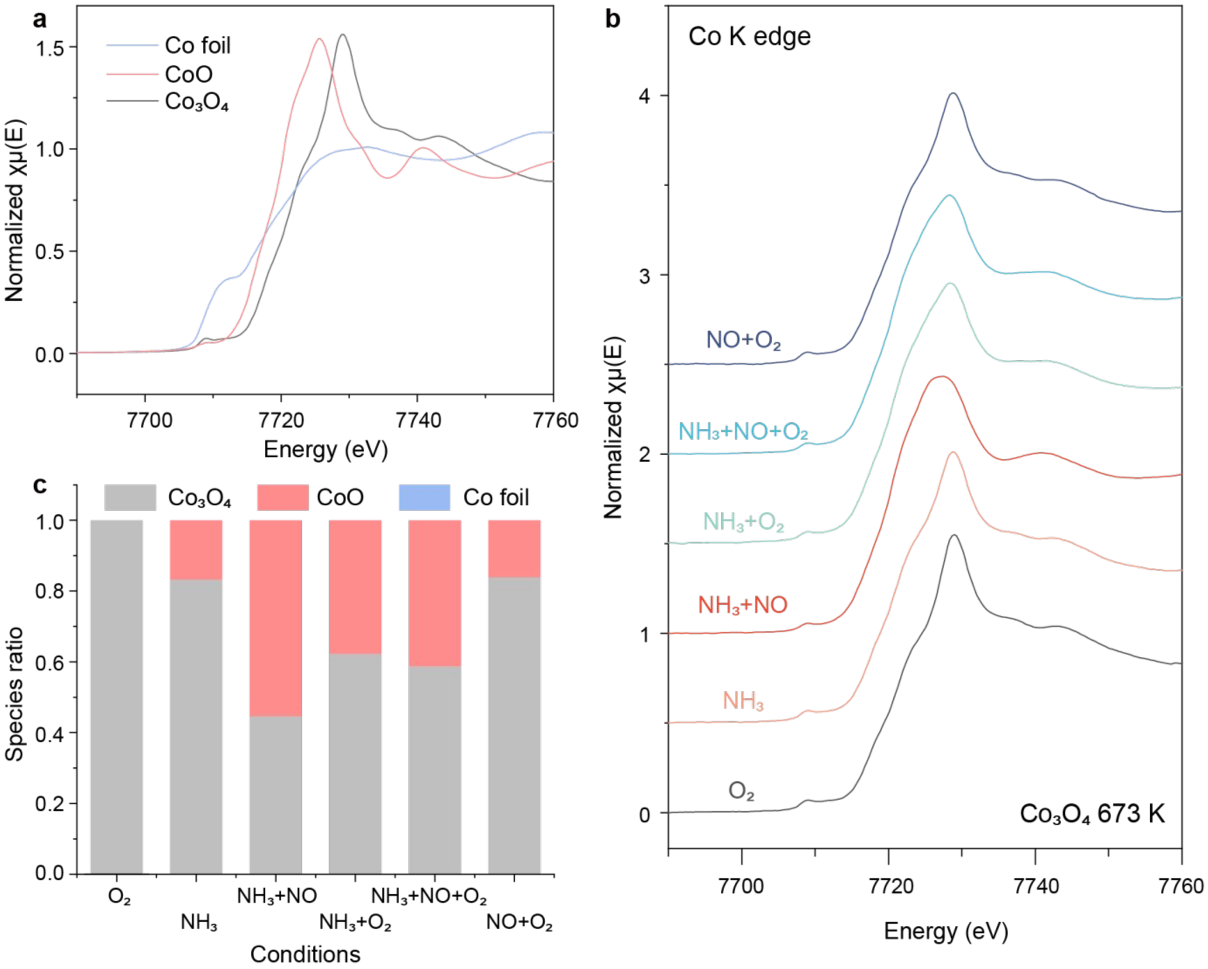

Figure S26. a, Co K edge XANES spectra of Co foil (blue), CoO standard (red) and Co_3O_4 standard (black). **b,** *In situ* XANES spectra of Co_3O_4 under various gas conditions at 673 K. **c,** Co speciation of Co_3O_4 catalyst under O_2, NH_3, NH_3+NO, NH_3+NO+O_2, NH_3+O_2, NO+O_2 conditions at 673 K.

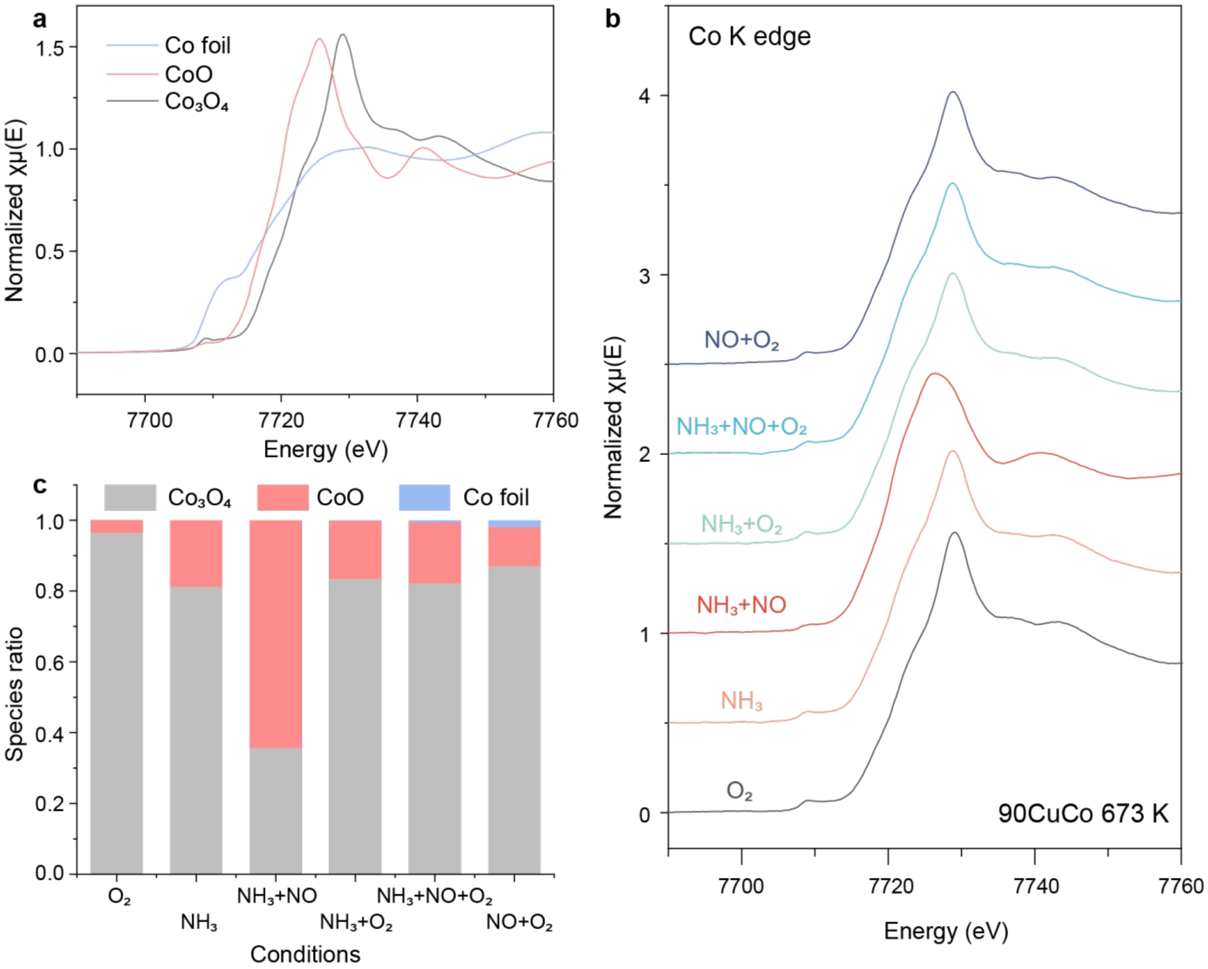

Figure S27. a, Co K edge XANES spectra of Co foil (blue), CoO standard (red) and Co_3O_4 standard (black). **b,** *In situ* XANES spectra of 90wt% CuO-Co_3O_4 under various gas conditions at 673 K. **c,** Co speciation of 90wt% CuO-Co_3O_4 catalyst under O_2, NH_3, NH_3+NO, NH_3+NO+O_2, NH_3+O_2, NO+O_2 conditions at 673 K.

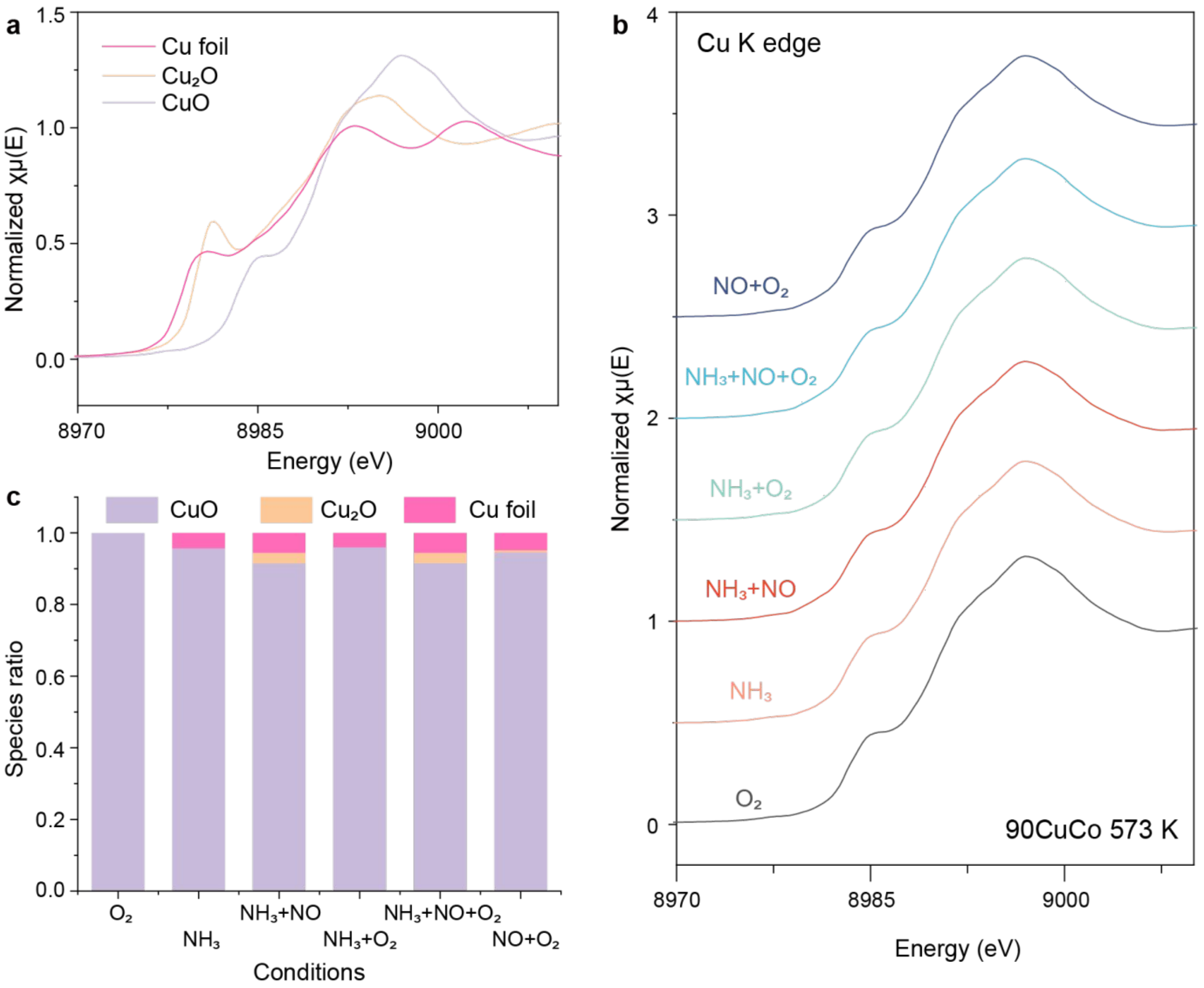

Figure S28. a, Cu K edge XANES spectra of Cu foil (pink), Cu_2O standard (orange) and CuO standard (purple). **b,** *In situ* XANES spectra of 90wt% CuO-Co_3O_4 under various gas conditions at 573 K. **c,** Cu speciation of 90wt% CuO-Co_3O_4 catalyst under O_2, NH_3, NH_3+NO, NH_3+NO+O_2, NH_3+O_2, NO+O_2 conditions at 573 K.

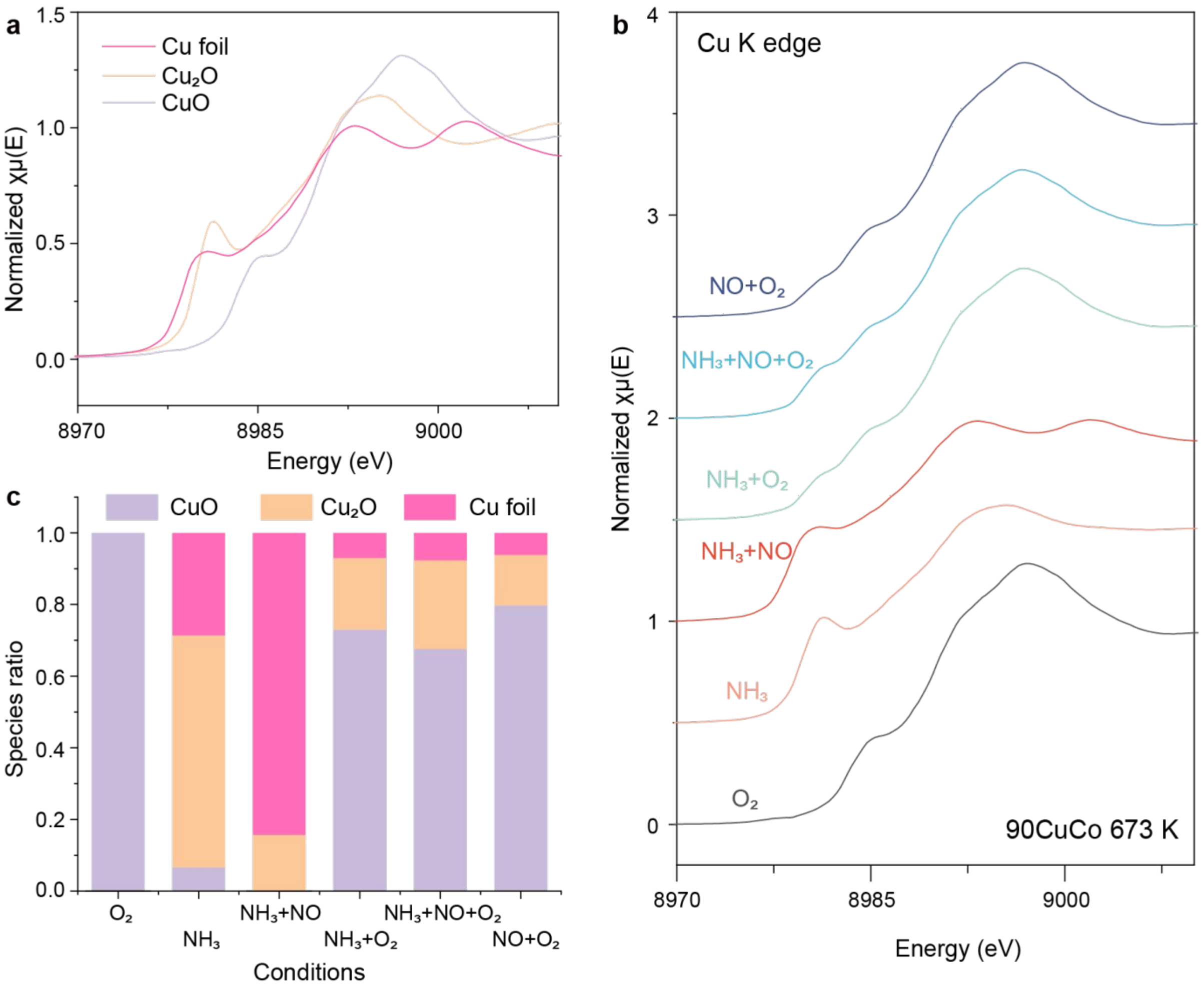

Figure S29. a, Cu K edge XANES spectra of Cu foil (pink), Cu_2O standard (orange) and CuO standard (purple). **b,** *In situ* XANES spectra of 90wt% CuO-Co_3O_4 under various gas conditions at 673 K. **c,** Cu speciation of 90wt% CuO-Co_3O_4 catalyst under O_2, NH_3, NH_3+NO, NH_3+NO+O_2, NH_3+O_2, NO+O_2 conditions at 673 K.

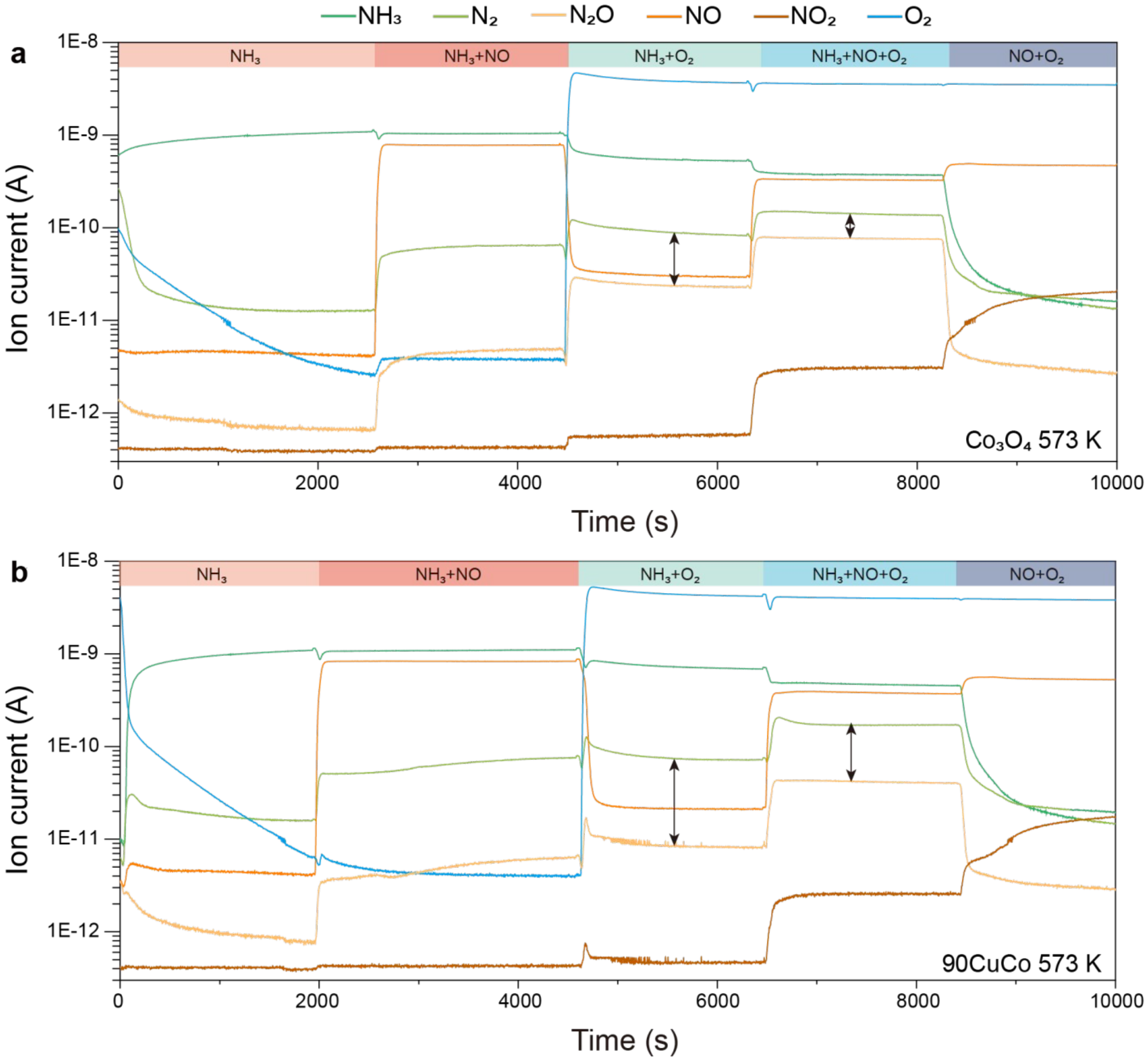

Figure S30. Mass spectrometry of *in situ* XAFS experiments for **a** Co_3O_4 and **b** 90CuCo under various gas conditions at 573 K.

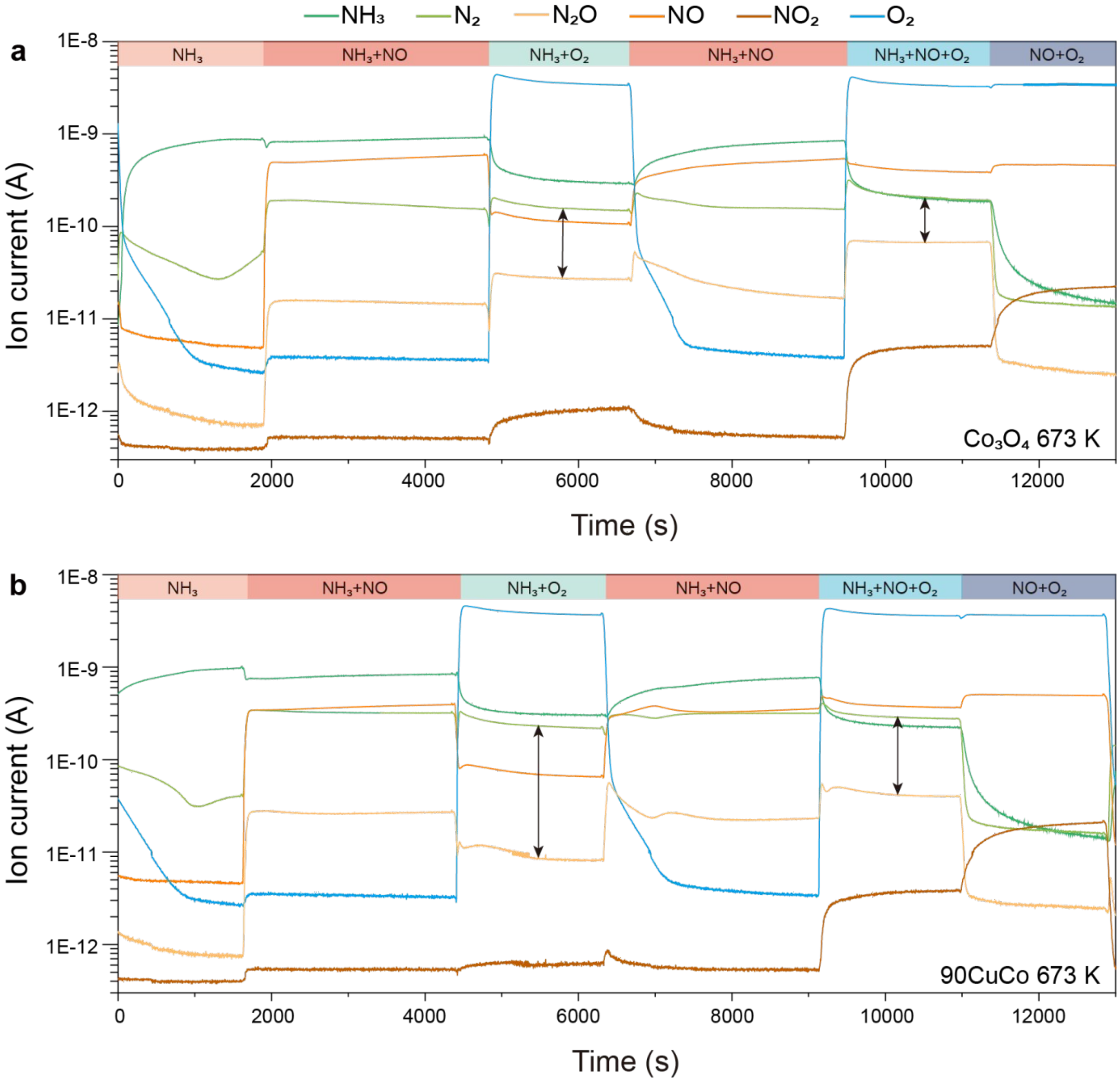

Figure S31. Mass spectrometry of *in situ* XAFS experiments for **a** Co_3O_4 and **b** 90CuCo under various gas conditions at 673 K.

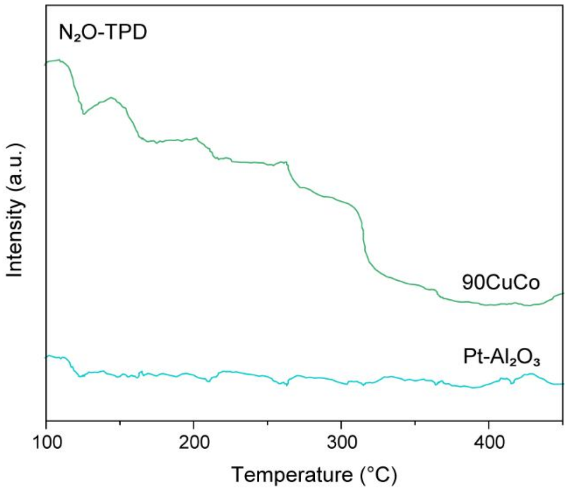

Figure S32. N_2O-TPD profiles of the Pt-Al_2O_3 and 90CuCo catalysts.

Table S1. Calculated NH_3, NO and O_2 adsorption energy on Co_3O_4 (100) surface.

Surface	Adsorbent	E_{surf} (eV)	$E_{surf+mol}$ (eV)	E_{ad} (eV)
Co_3O_4 (100)	NH_3	-725.40	-747.28	-2.34
	NO		-735.77	1.93
	O_2		-740.53	-5.27

Table S2. EXAFS fitting results of Cu K-edge in CuO-Co_3O_4 catalysts.

Sample	Scattering	C.N.	d (Å)	σ^2	E_0 (eV)
Cu foil STD	Cu-Cu	12	2.56		
Cu_2O STD	Cu-O	2	1.85		
	Cu-Cu	12	3.01		
CuO STD	Cu-O	4	1.95		
	Cu-Cu	4	2.88		
		4	3.07		
		2	3.16		
25wt% CuO-Co_3O_4	Cu-O	3.94 ± 0.26	1.96 ± 0.01	0.007 ± 0.001	-0.28 ± 0.70
	Cu-Cu (1)	4.94 ± 1.94	2.91 ± 0.02	0.014 ± 0.004	
	Cu-Cu (2)	3.42 ± 1.00	3.09 ± 0.02		
90wt% CuO- Co_3O_4	Cu-O	3.84 ± 0.44	1.96 ± 0.01	0.004 ± 0.001	0.57 ± 1.23
	Cu-Cu (1)	4.46 ± 1.41	2.90 ± 0.02	0.007 ± 0.002	
	Cu-Cu (2)	4.30 ± 1.47	3.09 ± 0.02		

Table S3. EXAFS fitting results of Co K-edge in CuO-Co_3O_4 catalysts.

Sample	Scattering	C.N.	d (Å)	σ^2	E_0 (eV)
Co foil STD	Co-Co	12	2.51		
CoO STD	Co-O	6	2.13		
	Co-Co	12	3.02		
Co_3O_4 STD	Co-O	6	1.92		
	Co-Co (1)	6	2.87		
	Co-Co (2)	6	3.37		
1wt% CuO-Co_3O_4	Co-O	5.25 ± 0.39	1.92 ± 0.01	0.003 ± 0.001	-4.38 ± 0.66
	Co-Co (1)	6.86 ± 0.66	2.88 ± 0.02	0.007 ± 0.001	
	Co-Co (2)	7.82 ± 0.76	3.38 ± 0.03		
25wt% CuO-Co_3O_4	Co-O	3.89 ± 0.25	1.92 ± 0.01	0.002 ± 0.001	-7.39 ± 0.54
	Co-Co (1)	4.59 ± 0.34	2.87 ± 0.01	0.005 ± 0.001	
	Co-Co (2)	4.53 ± 0.37	3.38 ± 0.03		
90wt% CuO-Co_3O_4	Co-O	4.25 ± 0.47	1.92 ± 0.01	0.004 ± 0.001	-3.64 ± 1.08
	Co-Co (1)	5.33 ± 0.90	2.87 ± 0.02	0.007 ± 0.001	
	Co-Co (2)	3.46 ± 0.67	3.38 ± 0.04		

Table S4. Review of catalytic performance of different catalysts for NH_3-SCO.

Catalysts	T (K)	NH_3 conversion (%)	N_2 selectivity (%)	WHSV ($ml_{NH3} \cdot h^{-1} \cdot g^{-1}$)
90wt%CuO-Co_3O_4 (This work)	553	100	96	120
	673	100	98	120
10wt%CuO-Al_2O_3[8]	623	100	93	30
30wt%CuO-RuO_2[10]	483	100	99	75
1.5%Ag-10% Cu/Al_2O_3[7]	648	100	94	120
CuO/CNTs (10 wt.%)[11]	462	100	98.7	60
10wt%Cu/TiO_2[12]	523	100	95	60

References

1. Wang, H.; Murayama, T.; Lin, M.; Sakaguchi, N.; Haruta, M.; Miura, H.; Shishido, T., Understanding the Distinct Effects of Ag Nanoparticles and Highly Dispersed Ag Species on N2 Selectivity in NH3–SCO Reaction. *ACS Catal.* **2022,** *12* (10), 6108-6118.
2. Sun, H.; Wang, H.; Qu, Z., Construction of CuO/CeO2 Catalysts via the Ceria Shape Effect for Selective Catalytic Oxidation of Ammonia. *ACS Catal.* **2023,** *13* (2), 1077-1088.
3. Xu, G.; Zhang, Y.; Lin, J.; Wang, Y.; Shi, X.; Yu, Y.; He, H., Unraveling the Mechanism of Ammonia Selective Catalytic Oxidation on Ag/Al2O3 Catalysts by Operando Spectroscopy. *ACS Catal.* **2021,** *11* (9), 5506-5516.
4. Qi, G. S.; Gatt, J. E.; Yang, R. T., Selective catalytic oxidation (SCO) of ammonia to nitrogen over Fe-exchanged zeolites prepared by sublimation of FeCl3. *J. Catal.* **2004,** *226* (1), 120-128.
5. Cui, X. Z.; Chen, L. S.; Wang, Y. X.; Chen, H. R.; Zhao, W. R.; Li, Y. S.; Shi, J. L., Fabrication of Hierarchically Porous RuO2-CuO/Al-ZrO2 Composite as Highly Efficient Catalyst for Ammonia-Selective Catalytic Oxidation. *ACS Catal.* **2014,** *4* (7), 2195-2206.
6. Jablonska, M.; Krol, A.; Kukulska-Zajac, E.; Tarach, K.; Chmielarz, L.; Gora-Marek, K., Zeolite Y modified with palladium as effective catalyst for selective catalytic oxidation of ammonia to nitrogen. *J. Catal.* **2014,** *316*, 36-46.
7. Jablonska, M.; Beale, A. M.; Nocun, M.; Palkovits, R., Ag-Cu based catalysts for the selective ammonia oxidation into nitrogen and water vapour. *Appl. Catal. B-Environ.* **2018,** *232*, 275-287.
8. Liang, C. X.; Li, X. Y.; Qu, Z. P.; Tade, M.; Liu, S. M., The role of copper species on Cu/gamma-Al2O3 catalysts for NH3-SCO reaction. *Appl. Surf. Sci.* **2012,** *258* (8), 3738-3743.
9. Lippits, M. J.; Gluhoi, A. C.; Nieuwenhuys, B. E., A comparative study of the selective oxidation of NH3 to N2 over gold, silver and copper catalysts and the effect of addition of Li2O and CeOx. *Catal. Today* **2008,** *137* (2), 446-452.
10. Cui, X. Z.; Zhou, J.; Ye, Z. Q.; Chen, H. R.; Li, L.; Ruan, M. L.; Shi, J. L., Selective catalytic oxidation of ammonia to nitrogen over mesoporous CuO/RuO2 synthesized by co-nanocasting-replication method. *J. Catal.* **2010,** *270* (2), 310-317.
11. Song, S. Q.; Jiang, S. J., Selective catalytic oxidation of ammonia to nitrogen over CuO/CNTs: The promoting effect of the defects of CNTs on the catalytic activity and selectivity. *Appl. Catal. B-Environ.* **2012,** *117*, 346-350.
12. He, S. L.; Zhang, C. B.; Yang, M.; Zhang, Y.; Xu, W. Q.; Cao, N.; He, H., Selective catalytic oxidation of ammonia from MAP decomposition. *Sep. Purif. Technol.* **2007,** *58* (1), 173-178.
